# Supplementary material for: Graphene collage on Ni-rich layered oxide cathodes for advanced lithium-ion batteries
Source: Nat Commun. 2021 Apr 9;12:2145. doi: 10.1038/s41467-021-22403-w (PMC8035182; doi:10.1038/s41467-021-22403-w)
Supplement: Supplementary file 1 — Supplementary information [file 41467_2021_22403_MOESM1_ESM.docx]

*Supplementary information*

**Graphene collage on Ni-rich layered oxide cathodes for advanced lithium-ion batteries**

Chang Won Park^1,2†^, Jung-Hun Lee^3,†^, Jae Kwon Seo^3^, Won Young Jo^3^, Dongmok Whang^1,3^, Soo Min Hwang^3,^*, and Young-Jun Kim^3,4,^*

*^1^ School of Advanced Materials Science and Engineering, Sungkyunkwan University, Suwon 16419, Republic of Korea*

*^2^ Samsung SDI Co., LTD, Suwon 16678, Republic of Korea*

*^3^ SKKU Advanced Institute of Nano Technology (SAINT), Sungkyunkwan University, Suwon 16419, Republic of Korea*

*^4^ Department of Nano Engineering, Sungkyunkwan University, Suwon, 16419, Republic of Korea*

^†^These authors contributed equally to this work.

*Corresponding authors: [smhwang@skku.edu](mailto:smhwang@skku.edu) (S.M. Hwang); [yjkim68@skku.edu](mailto:yjkim68@skku.edu) (Y.-J. Kim)


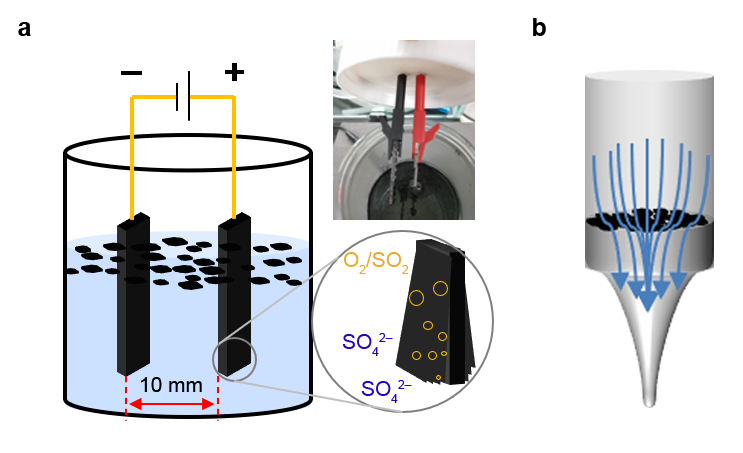


**Supplementary Fig. 1| Electrochemical exfoliation of graphite foils.** **a**, Experimental set-up of electrochemical exfoliation of graphite foils. Two graphite foils are immersed in an aqueous solution of 0.5 M ammonium persulfate ((NH_4_)_2_S_2_O_8_), in parallel at a fixed distance of 10 mm. When the graphite foils are biased (+10 V), OH^−^ and SO_4_^2−^ ions in the electrolyte are intercalated into the graphitic layers, followed by the evolution of gases, such as SO_2_ and O_2_, which makes the foils exfoliated into Gr nanosheets^1^. **b**, The produced Gr nanosheets are collected by vacuum filtration and washing process.


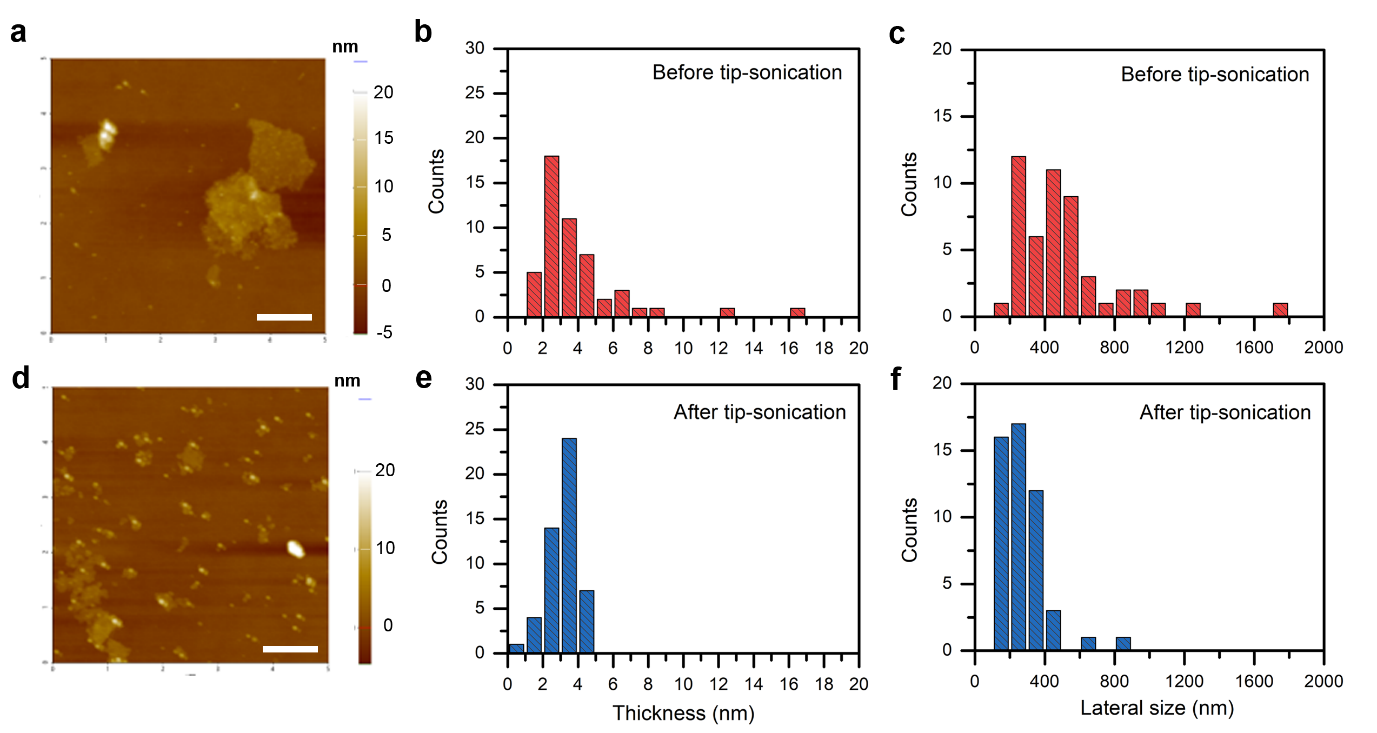


**Supplementary Fig. 2|** AFM images (5 μm × 5 μm; **a**,**d**) and size-/thickness-distributions (histograms; **b**,**c**,**e**,**f**) of exfoliated Gr sheets before (**a**-**c**) and after tip-sonication (**d**-**f**). The scale bars in **a**,**d** indicate 1 μm.

**

**

**Supplementary Fig. 3|** XPS C 1*s* spectra of as-exfoliated Gr (top) and DSPE-mPEG-functionalized Gr nanosheets (bottom).


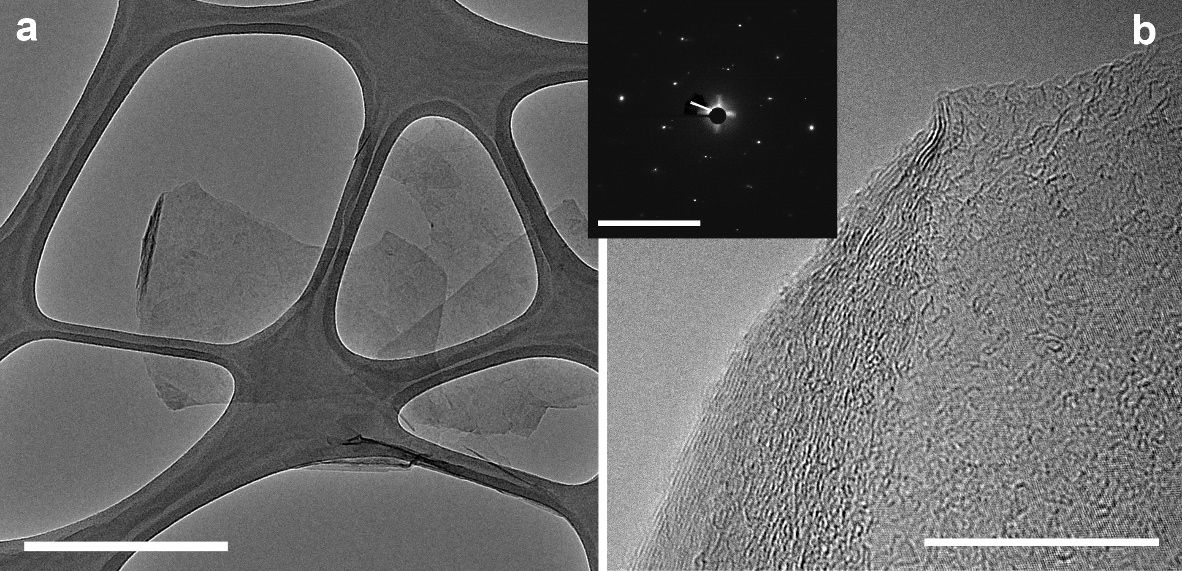


**Supplementary Fig. 4|** TEM images and SAED pattern (inset) of DSPE-mPEG-functionalized Gr nanosheets. The scale bar in **a** indicates 500 nm. The interplanar spacing in **b** was measured to be ~0.35 nm, corresponding to (00*l*) plane of Gr. The scale bars in **b** and the inset indicate 20 nm and 1/10 nm, respectively.


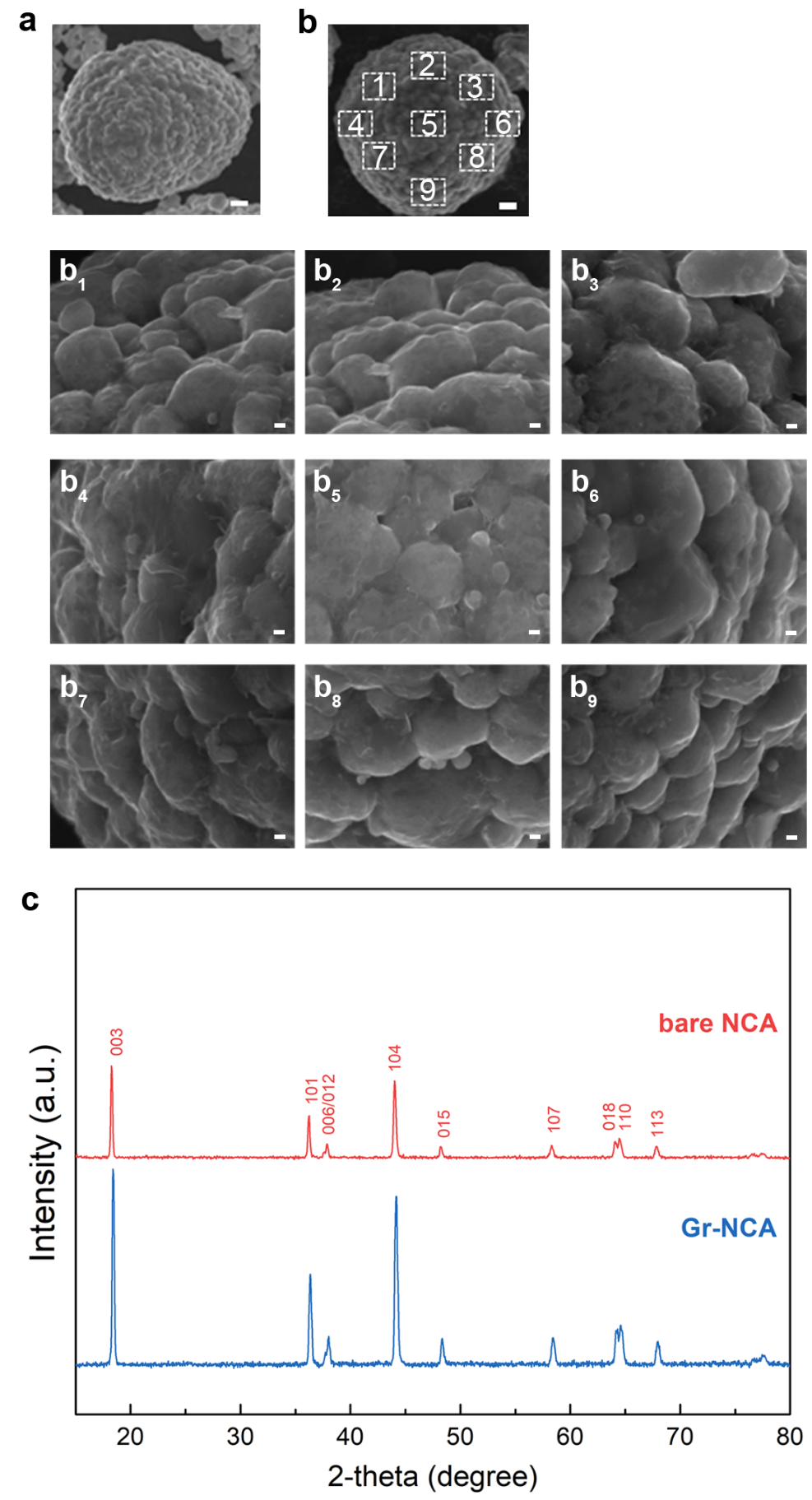


**Supplementary Fig. 5|** SEM images of (**a**) bare and (**b** and **b_1_–b_9_**) Gr-coated (thrice) NCA particles and (**c**) XRD patterns. The scale bars in **a**,**b** indicate 1 μm, and the scale bars in **b_1_**–**b_9_** indicate 100 nm.

**
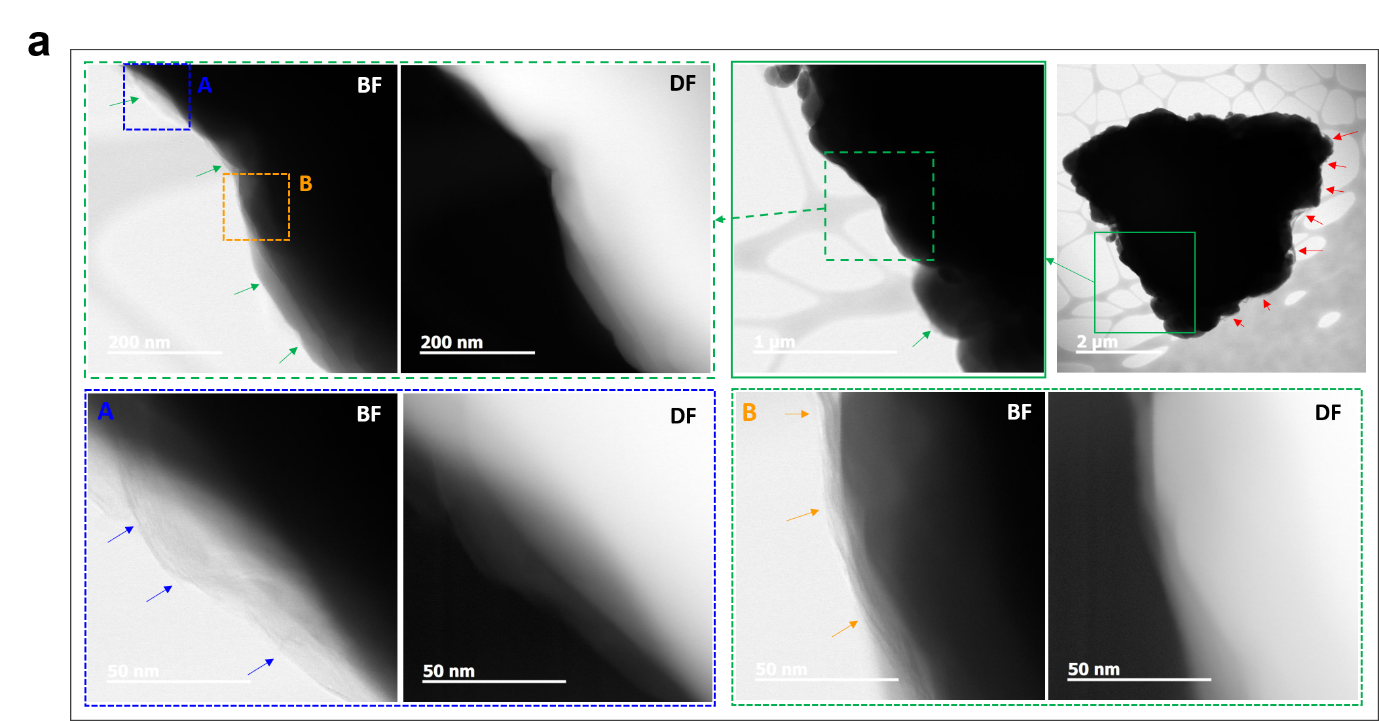
**

**
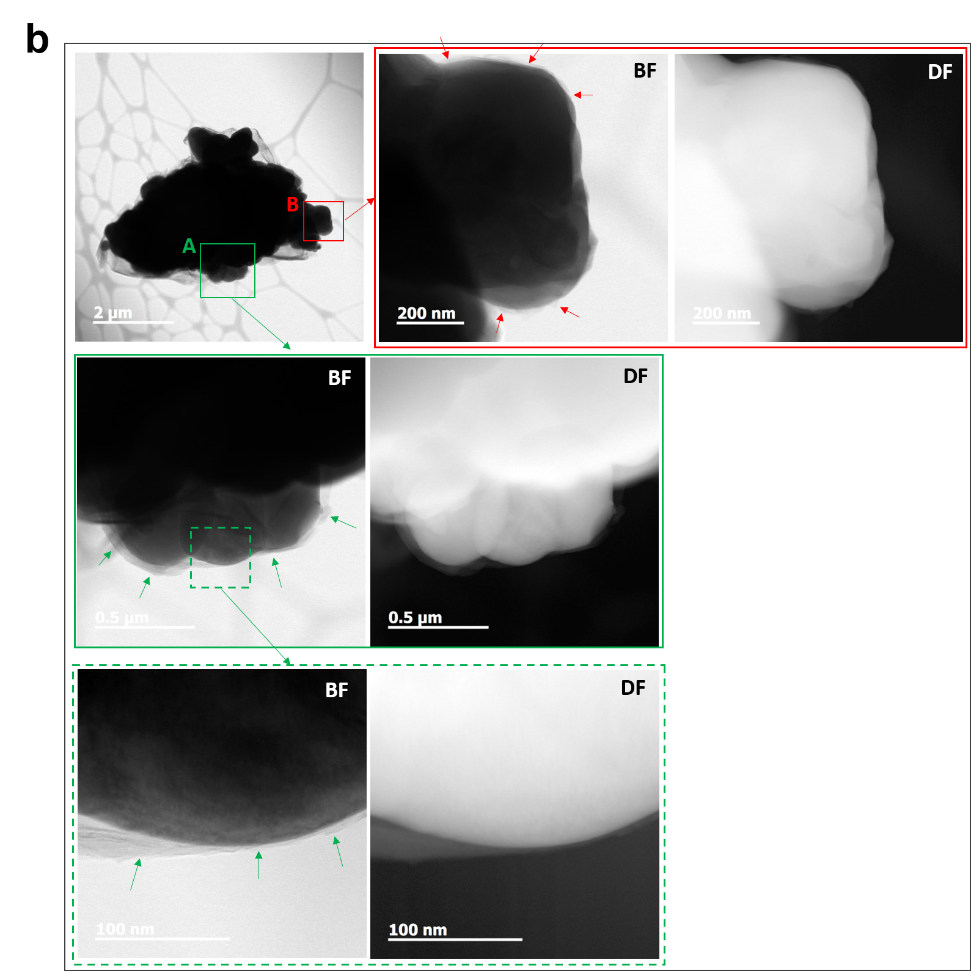
**

(Continued)

**
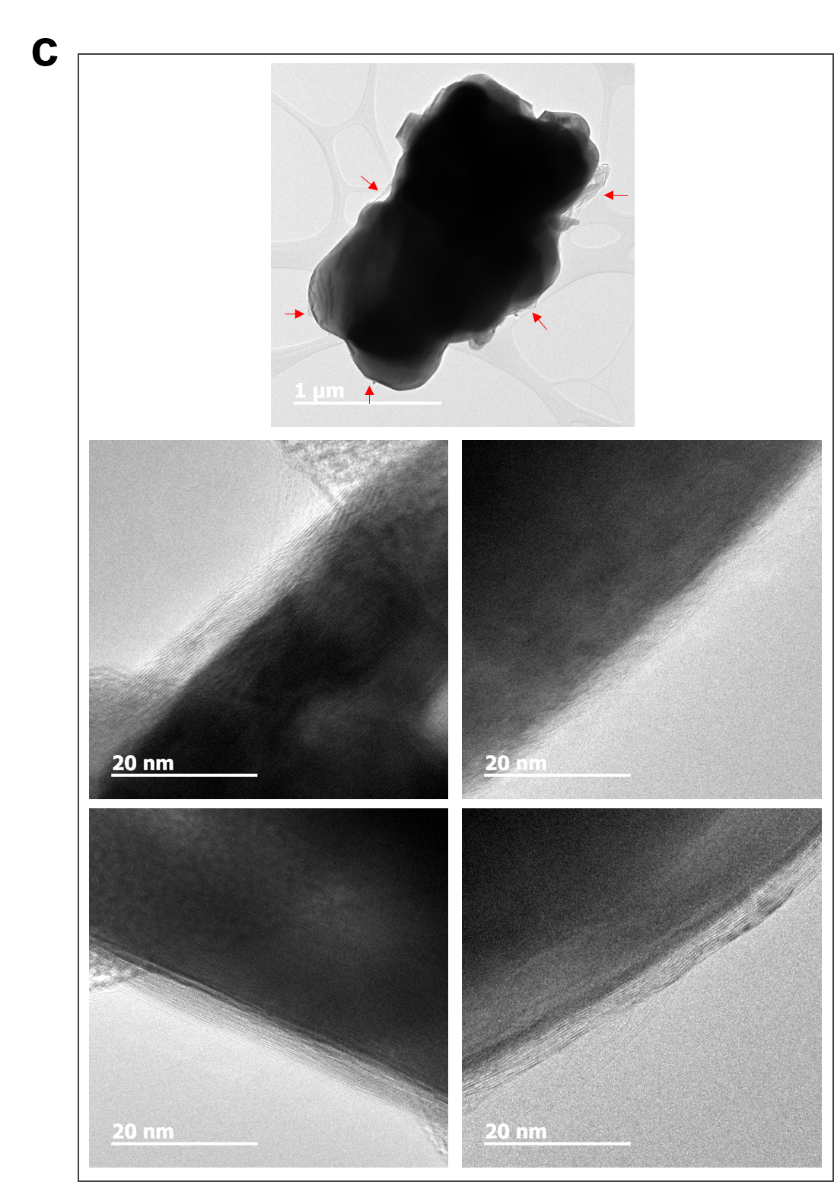
**

(Continued)


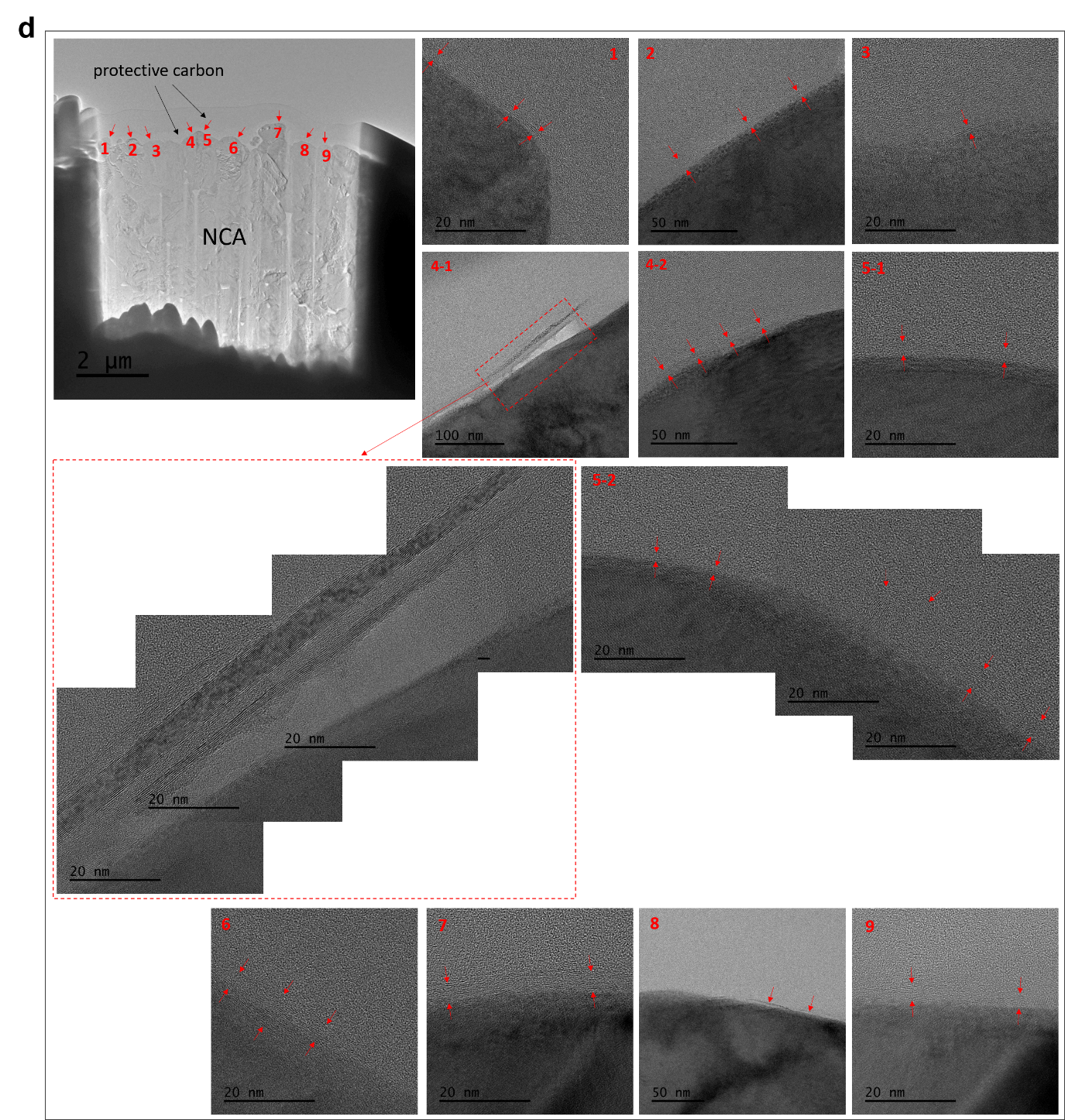


**Supplementary Fig. 6|** **Collection of TEM micrographs showing the Gr coverage on NCA particles**. TEM images of Gr-coated (thrice) NCA particles (**a**-**c**) and cross-sectioned particle using FIB (**d**). The red arrows highlight the presence of Gr nanosheets attached on the surface of NCA.


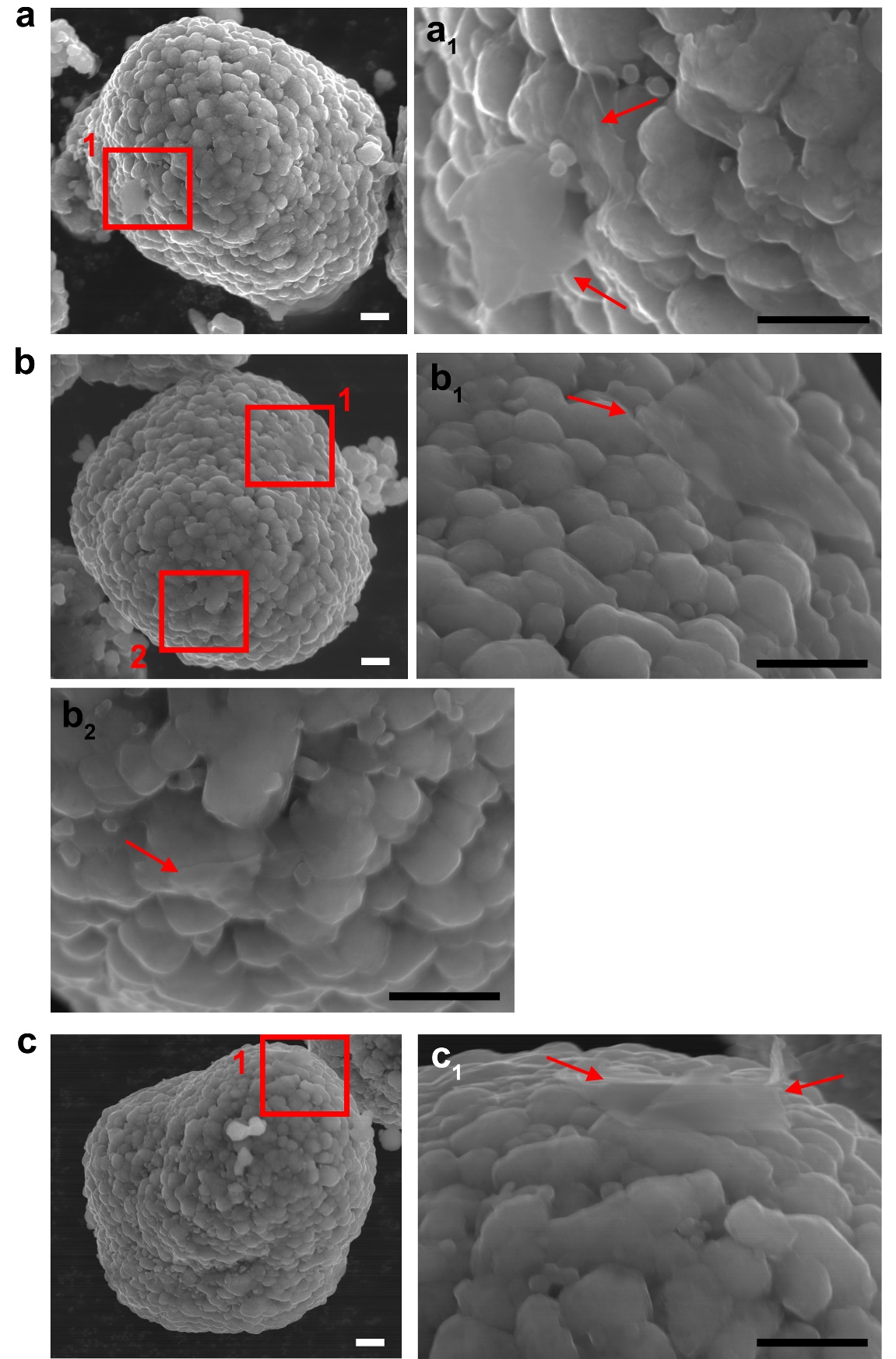


**Supplementary Fig. 7|** SEM images of NCA particles subjected to Gr-coating process (thrice) without the use of DSPE-mPEG surfactant. The arrows highlight Gr nanosheets which are attached locally on the NCA surface. Some of the Gr nanosheets are observed to be not attached in a face-to-face manner. All the scale bars indicate 1 μm.





**Supplementary Fig. 8|** TGA graphs of bare and Gr-coated (thrice) NCA particles. The inset shows the TGA graph of functionalized Gr. The Gr content in coated NCA particles is estimated to be ~0.5 wt% from the difference in the weight losses between the bare and Gr-coated NCA samples at 820 °C.

**Supplementary Note 1. Scaling of the measured conductivity with percolation theory**

For composites containing conducting agents, the electrical conductivity is described by percolation theory. Based on this model, the conductivity increases only above a critical volume fraction where the first complete conductive pathway is formed, i.e., a value known as the percolation threshold, *ϕ*_c_. Above this threshold, the composite conductivity, σ, scales as follows^2,3^:

$$\sigma=\sigma_{0}\left( \emptyset-\emptyset_{c} \right)^{n},for \emptyset>\emptyset_{c}$$

where σ_0_ is a constant related to the conductivity of the filler network and *n* is the percolation exponent.





**Supplementary Fig. 9|** N_2_ adsorption/desorption isotherms of bare NCA, Gr-coated NCA, and mixed (NCA+CB) powders and pore-size distribution (inset) calculated by the Barrett–Joyner–Halenda (BJH) method.





**Supplementary Fig. 10| FT-IR spectra of bare and Gr-coated NCA powders.** For comparison, the FT-IR of DSPE-mPEG is shown. The Gr-coated powders show the stretching vibrations of sp^3^-CH_3_ and phosphate-containing groups, such as P=O and -P–O–C-, implying the presence of DSPE-mPEG in the coated particles^4-6^.


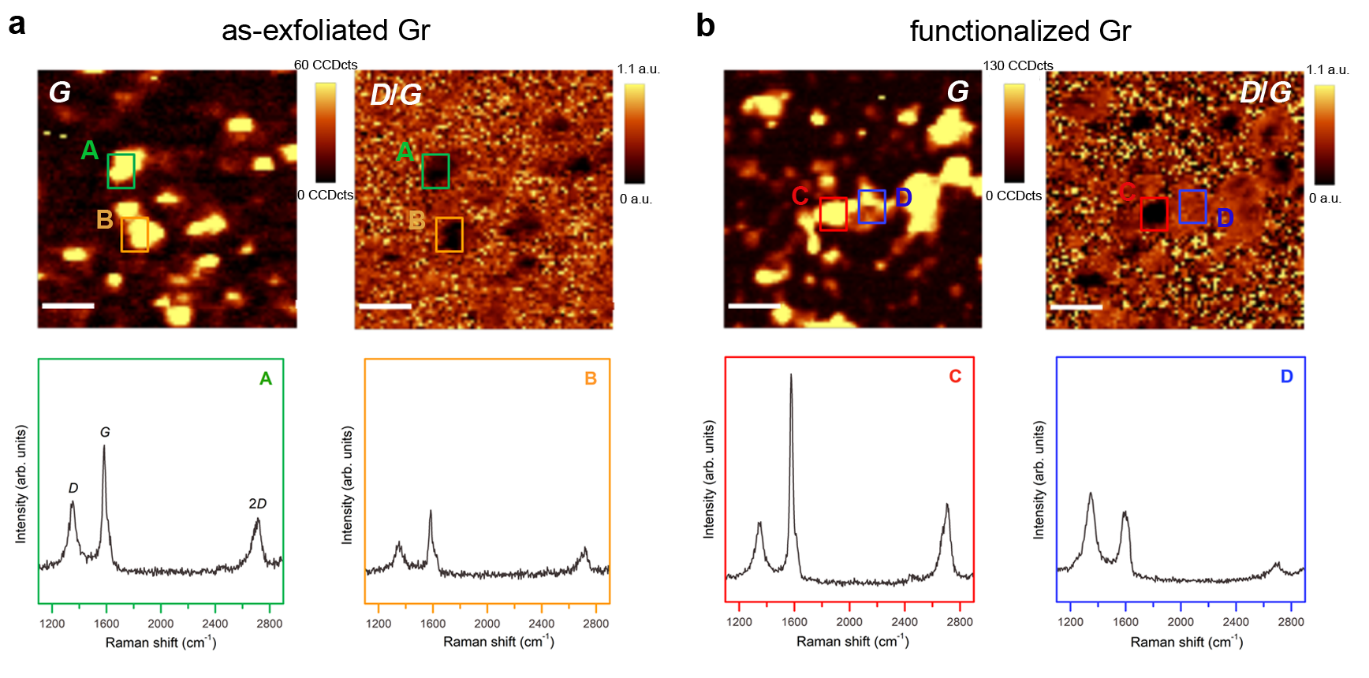


**Supplementary Fig. 11|** Raman mapping images (top) and spectra (bottom) of as-exfoliated Gr nanosheets (**a**) and DSPE-mPEG-functionalized Gr nanosheets (**b**). All the scale bars indicate 4 μm. The Raman mapping images show the *G* band and relative intensity from *D* to *G* band. The functionalized Gr nanosheets locally have high *D*/*G* regions, in comparison with the as-exfoliated Gr nanosheets, probably due to the presence of DSPE-mPEG attached on the Gr surface.


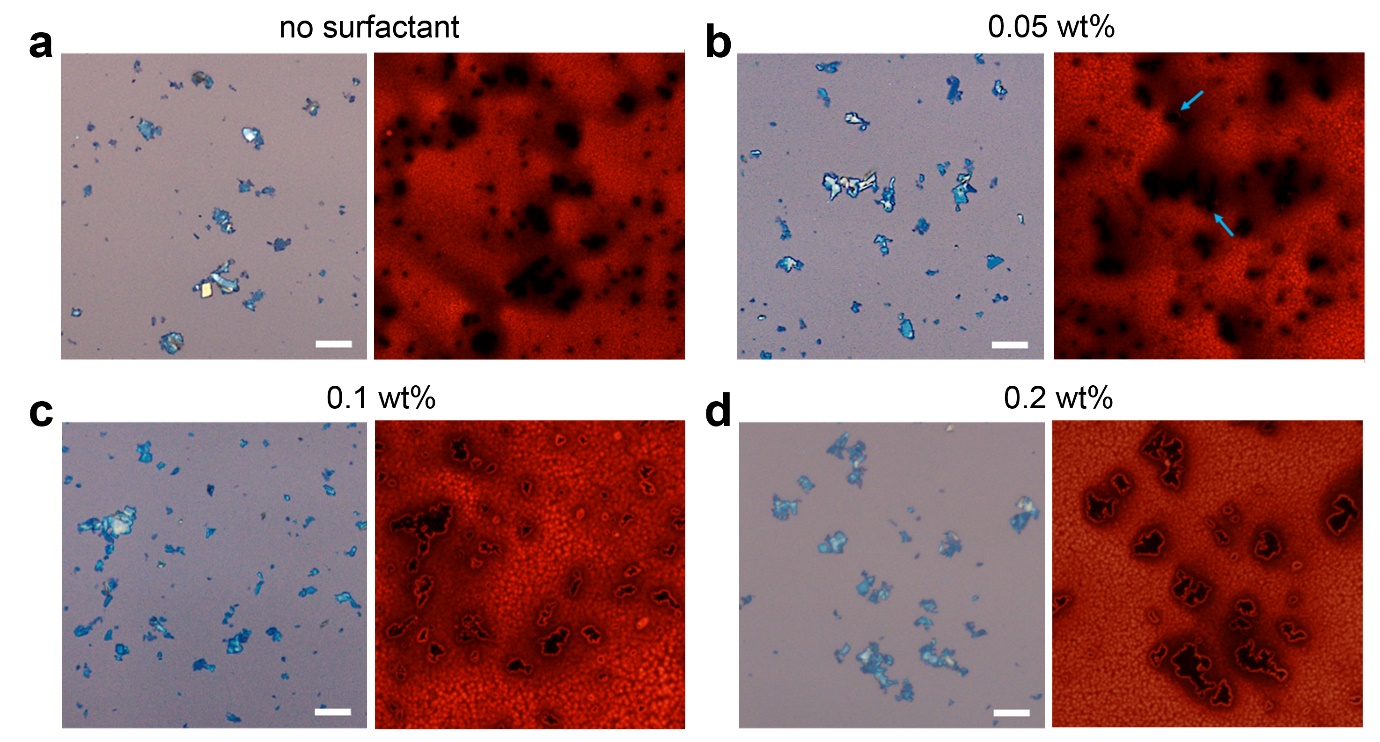


**Supplementary Fig. 12| FM imaging of as-exfoliated Gr and functionalized Gr nanosheets.** Optical (left) and FM (right) images of (**a**) as-exfoliated Gr and (**b**-**d**) functionalized Gr nanosheets with different DSPE-mPEG contents (0.05-0.2 wt%) on SiO_2_/Si wafer. Rhodamine B was used as fluorescence dye for FM imaging. The dark regions are due to the absence of the dye or the quenching by Gr sheets. The light blue arrows in **b** indicate the trace of fluorescence near the Gr edges. All the scale bars indicate 10 μm.


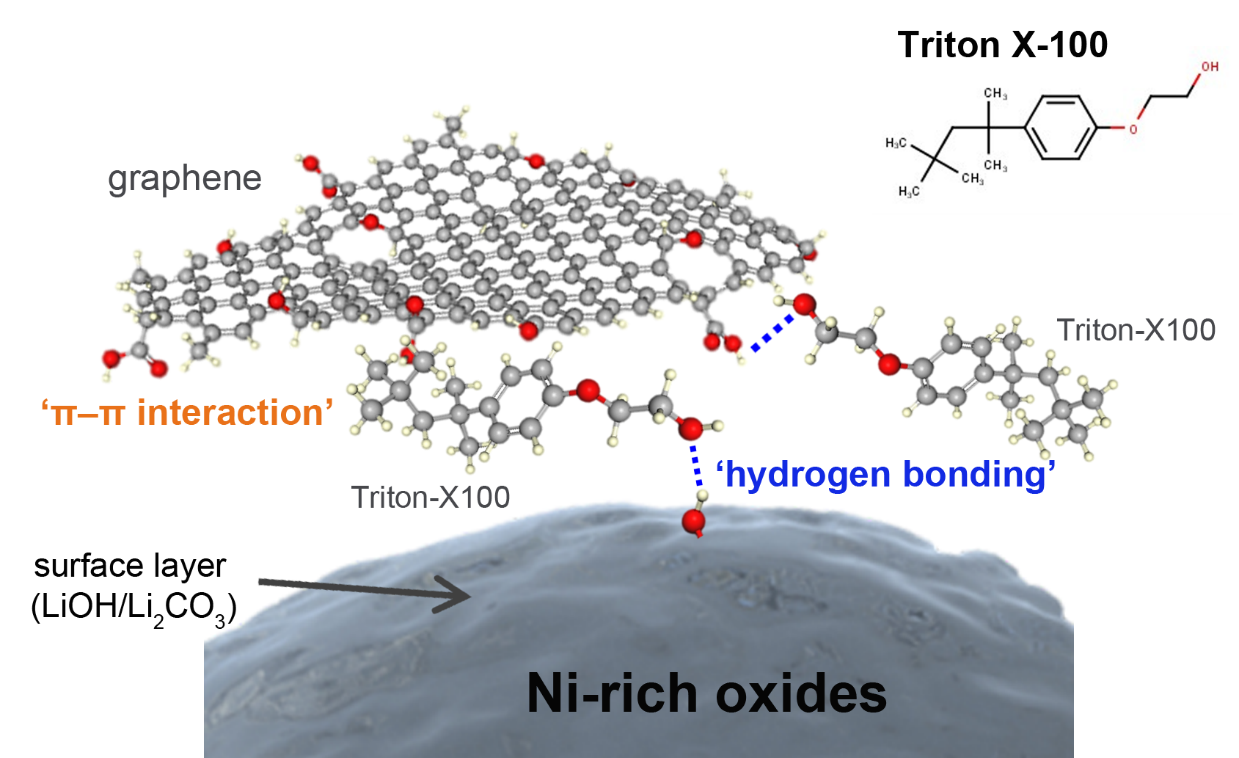


**Supplementary Fig. 13| Schematic of the bonding configuration of Triton X-100 between Gr nanosheets and Ni-rich oxide particles.** The surfactant and Gr layer are illustrated using a ball-and-stick model (drew by Marvin Sketch), where red, grey, and ivory indicate oxygen, carbon, and hydrogen, respectively. The amphiphilic surfactants bind to the oxide surface by their hydrophilic head (“hydrogen bonding” with the hydroxyl groups of oxide surface), while retaining “π–π interactions” between the basal plane of Gr and their hydrophobic tails. In addition, the surfactant could bind to Gr edges by the hydrophilic head via “hydrogen bonding” with the hydroxyl functional groups of Gr, consequently contributing to a conformal Gr coating in a face-to-face manner.

**Supplementary Note 2. Effect of surfactants on the Gr coating behavior**

Controlled experiments were conducted by varying the surfactant type, to investigate the effect of surfactants on the Gr coating behavior on NCA particles. We prepared Gr dispersions (1 wt% Gr in DMF) containing different types of surfactants/additives, such as Triton X-100 (C_14_H_22_O(C_2_H_4_O)*_n_*; non-ionic), sodium dodecyl sulfate (SDS, CH_3_(CH_2_)_11_SO_4_Na; anionic), cetyltrimethylammonium bromide (CTAB, [(C_16_H_33_)N(CH_3_)_3_]Br]; cationic), and polyvinylpyrrolidone (PVP). The use of Triton-X100 and PVP exhibited good dispersion of Gr, whereas the others induced agglomeration. The NCA particles were subjected to thrice-coating process using surfactants of Triton X-100, PVP, and DSPE-mPEG. The NCA particles coated using Triton X-100 exhibited good coverage of Gr, overall, which was comparable to the case of DSPE-mPEG (**Supplementary Fig. 14a,c**). In contrast, the NCA particles coated using PVP showed slightly poor coverage of Gr (**Supplementary Fig. 14b**). The resulting discharge rate capability of the Gr-coated NCA electrodes using those surfactants, measured in the voltage range of 2.75-4.3 V vs. Li^+^/Li at 0.2-5C in a half-cell configuration, is shown in **Supplementary Fig. 14d**. For the Triton-X, the electrode displays good rate performance, comparable to the electrode using DSPE-mPEG. This result partly supports our conjecture, since Trion-X is a non-ionic, amphiphilic surfactant that has a hydrophilic polyethylene oxide chain (“hydrogen bonding” part) and hydrophobic hydrocarbon group (“π–π interactions” part) (refer to **Supplementary Fig. 13**).

**
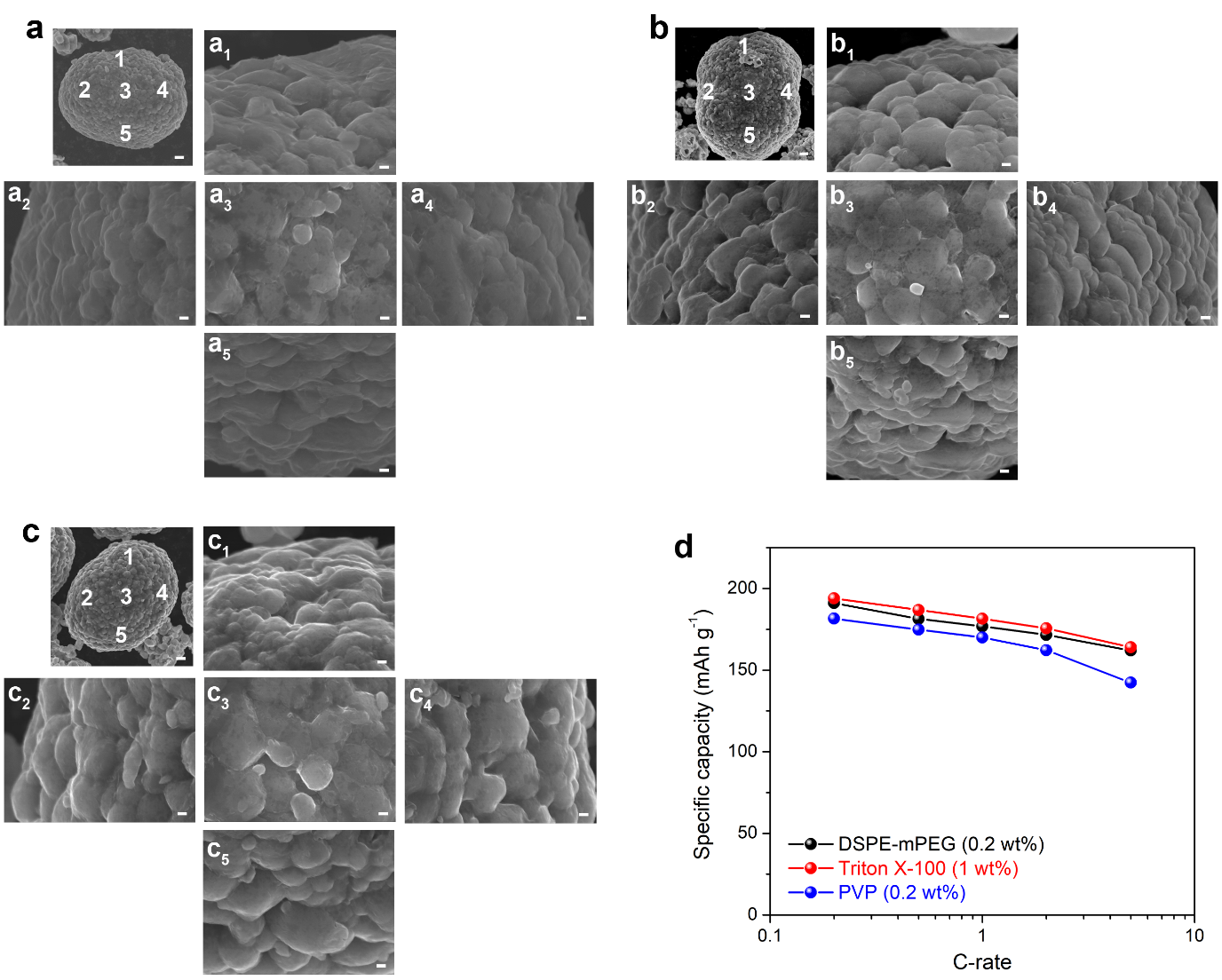
**

**Supplementary Fig. 14|** SEM images of Gr-coated (thrice) NCA particles (**a**,**b**,**c**) and rate capability of the coated electrodes measured at 0.2-5C (**d**). The Gr dispersions (1 wt% Gr) containing Triton X-100 (1 wt%) (**a**), PVP (0.2 wt%) (**b**), and DSPE-mPEG (0.2 wt%) (**c**) were used to coat NCA particles. The scale bars in **a**,**b**,**c** and **a_1_**-**a_5_**;**b_1_**-**b_5_**;**c_1_**-**c_5_** indicate 1 μm and 100 nm, respectively.

**Supplementary Note 3. Optimization of Gr-coating process and binder content**

We investigated the effects of the DSPE-mPEG content and Gr coating on the electrochemical properties of NCA cathodes in a half-cell configuration. The Gr-coated (1 time) NCA particles were prepared using Gr dispersions (1 wt% Gr) with different DSPE-mPEG contents (0-0.2 wt%) and then the electrodes were fabricated without the use of CB (NCA:CB:PVdF (φ) = 99.5:0:0.5). **Supplementary Fig. 15** shows the rate capability of the bare and Gr-coated NCA electrodes, whose *ρ* are set as 3.9±0.1 g cm^-3^ based on the powder conductivity data (**Fig. 1k**), enabled by the removal of CB and equivalent amount of PVdF. The bare electrode and coated electrode without the use of DSPE-mPEG show almost similar rate properties at 0.1-5C, although the coated one has a slightly higher capacity at low C-rates (**Supplementary Fig. 15f**). With an increase in the content of DSPE-mPEG upon Gr coating, the NCA electrodes show better rate performance: the optimal content is found to 0.1 wt%. These results suggest that the DSPE-mPEG plays a pivotal role in Gr coating on NCA particles; however, 1 time-coating appears to be insufficient for full coverage of Gr. Therefore, we repeated the Gr coating process. The rate capability of the electrodes subjected to different coating times at a fixed DSPE-mPEG content of 0.1 wt% is shown in **Supplementary Fig. 16**. The thrice-coating is optimal for NCA electrodes with φ = 99.5:0:0.5 (**Supplementary Fig. 16e**), which is comparable to the bare electrode with a commercial level of electrode parameters (φ = 96:2:2; *ρ* ~3.3 g cm^-3^). For PVdF content (**Supplementary Fig. 17**), the electrodes using 0.5-1 wt% PVdF display similar rate properties at 0.1-5C, whereas less content (<0.5 wt%) induces partial delamination of films from current collector (see the inset). These findings show that the coated Gr nanosheets form sufficient conductive percolation networks within the electrodes even without conventional conducting agents (CB).

**
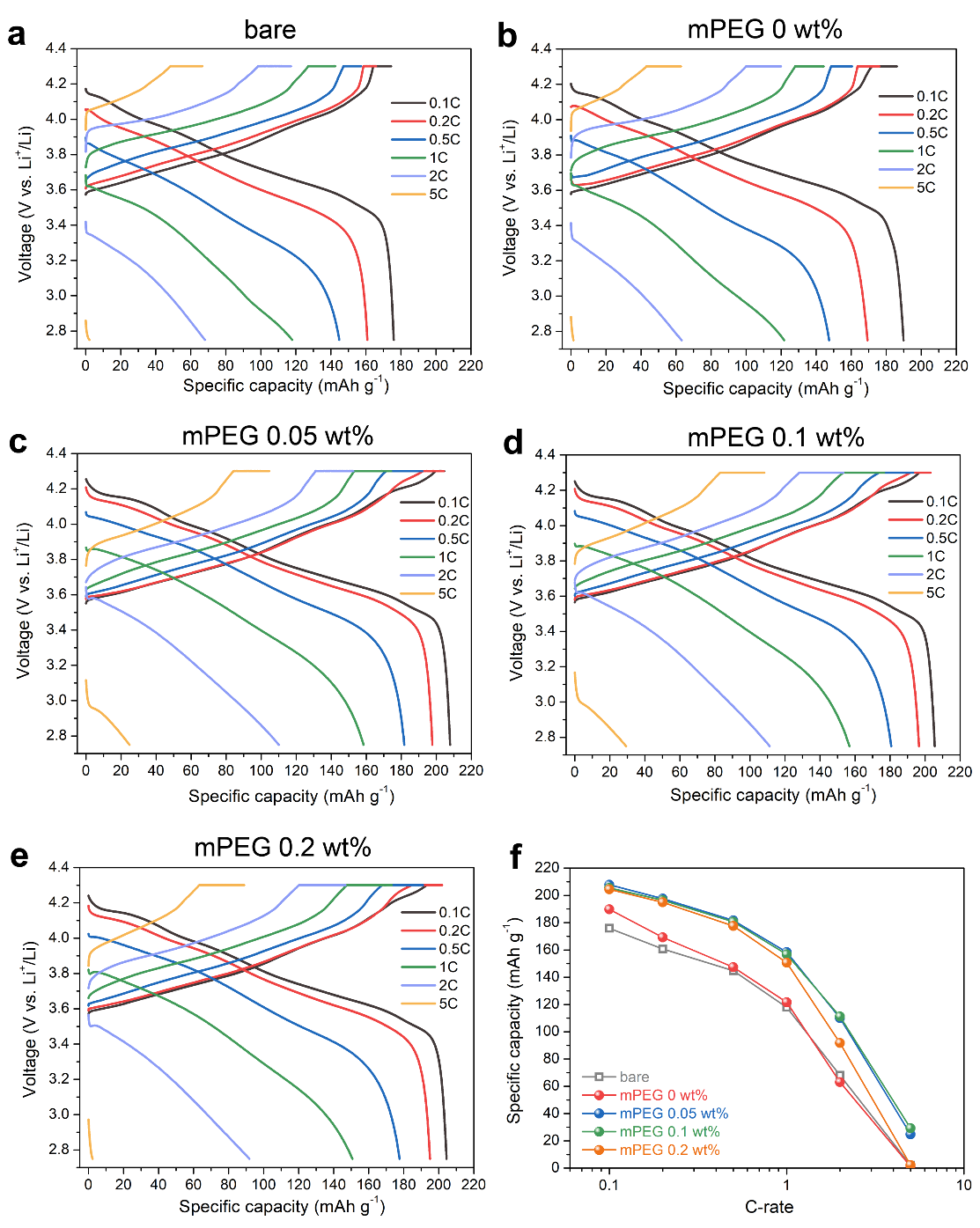
**

**Supplementary Fig. 15| Effect of DSPE-mPEG content on the rate capability of Gr-coated NCA cathodes**. **a**-**e**, Voltage profiles of bare electrode (**a**) and Gr-coated (1 time) electrodes with DSPE-mPEG contents of 0-0.2 wt% (**b**-**e**), measured at 30 °C in the voltage range of 2.75-4.3 V vs. Li^+^/Li at 0.1-5C. **f**, Comparison of the resulting rate capability. All the electrodes have electrode parameters of φ = 99.5:0:0.5 and *ρ* ~3.9 g cm^-3^.


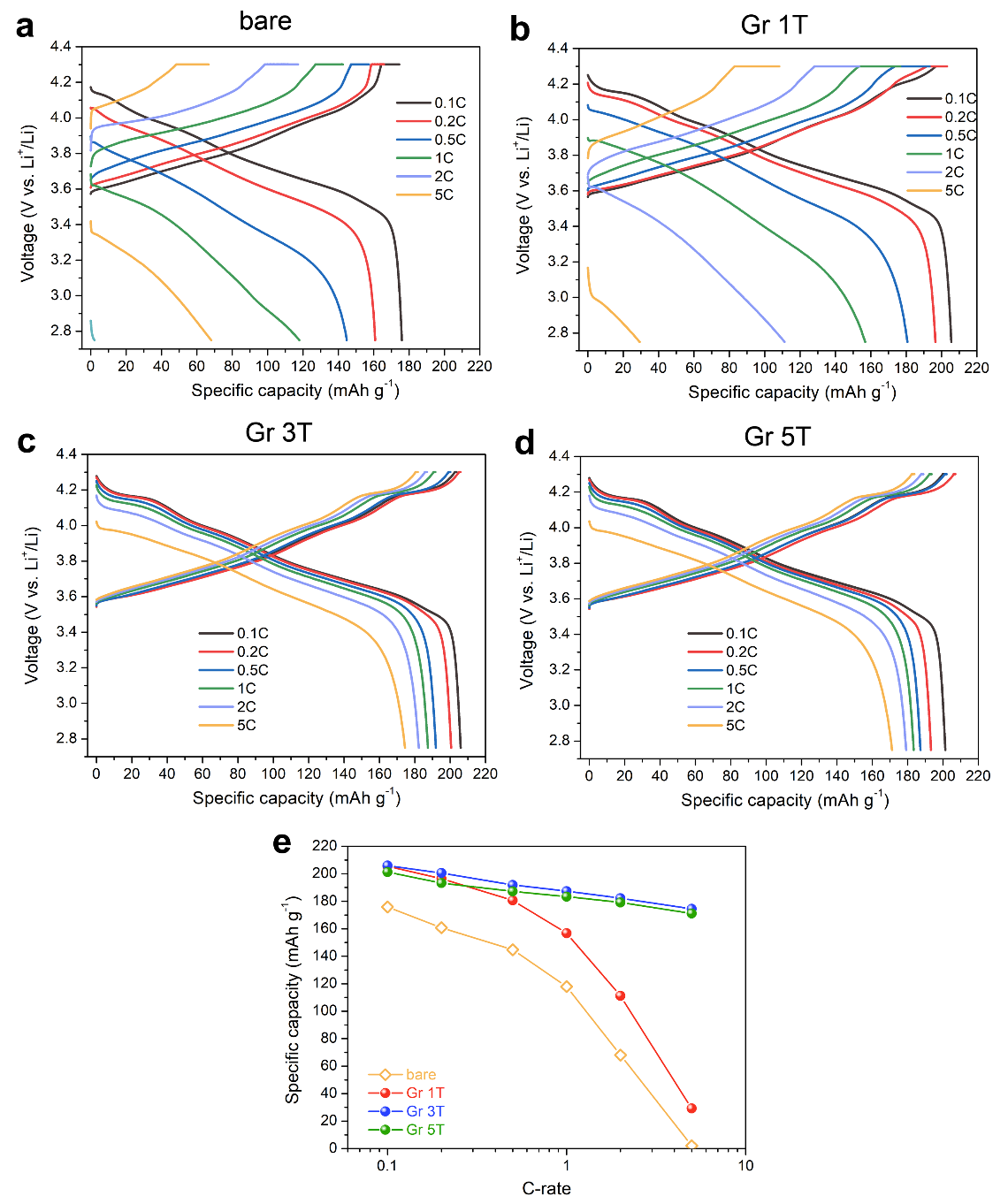


**Supplementary Fig. 16| Effect of multi-coating on the rate capability of Gr-coated NCA cathodes. a-d,** Voltage profiles of bare electrode (**a**) and Gr-coated electrodes with coating times (1-5 times) (**b**-**d**), measured at 30 °C in the voltage range of 2.75-4.3 V vs. Li^+^/Li at 0.1-5C. **e**, Comparison of the resulting rate capability. All the electrodes have electrode parameters of φ = 99.5:0:0.5 and *ρ* ~3.9 g cm^-3^.


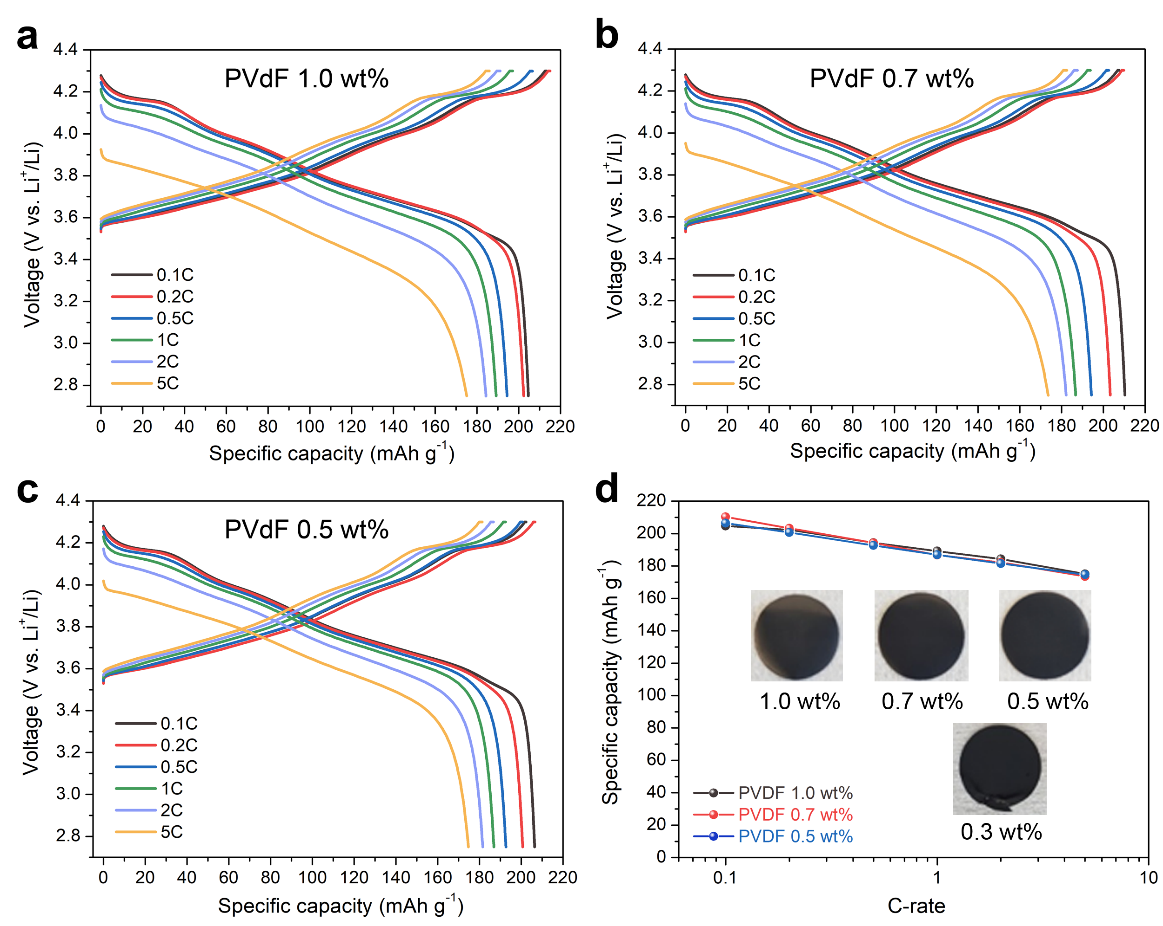


**Supplementary Fig. 17| Rate performance of the Gr-coated NCA electrodes with different PVdF contents**. **a-c,** Voltage profiles of Gr-coated (thrice) electrodes with PVdF contents of 0.5-1.0 wt%, measured at 30 ^o^C in the voltage range of 2.75-4.3 V vs. Li^+^/Li at 0.1-5C. **d**, Comparison of the resulting rate capability. All the electrodes have electrode parameters of φ = 100-*x*:0:*x* and *ρ* ~3.9 g cm^-3^. The inset in **d** shows the digital images of the Gr-coated electrodes with different PVdF contents. The electrode using 0.3 wt% PVdF exhibits the edge delamination of film.





**Supplementary Fig. 18|** Nyquist plots of bare and Gr-coated electrodes with different electrode parameters, measured before formation process.


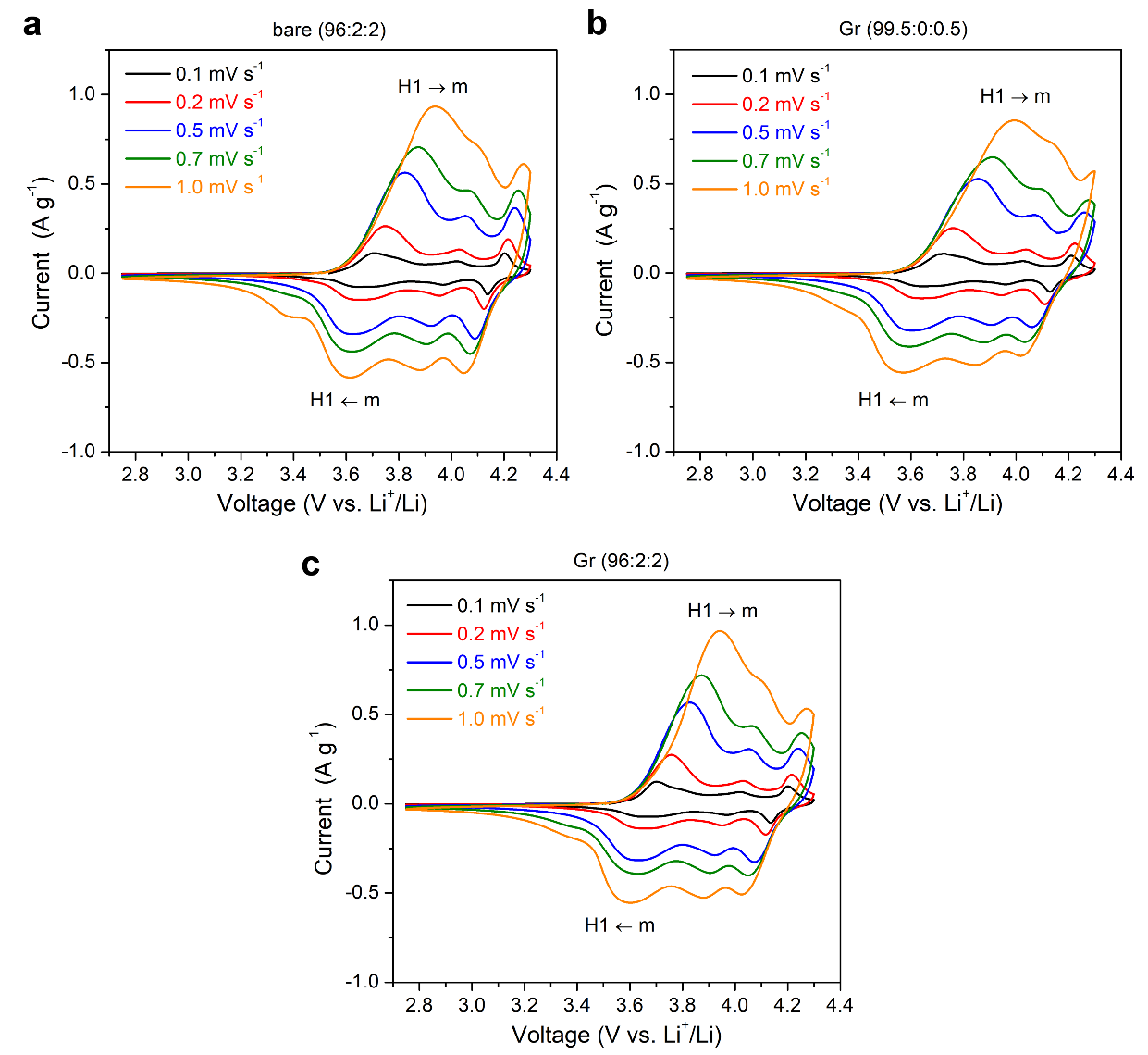


**Supplementary Fig. 19|** Cyclic voltammetry curves of bare (**a**) and Gr-coated (**b**,**c**) NCA electrodes, measured in the voltage range of 2.75-4.3 V vs. Li^+^/Li at scan rates of 0.1-1 mV s^-1^. The peak current (*i*_p_) was determined at peaks related to the phase transition between the hexagonal (H1) and monoclinic (m) structures. The bare electrode (**a**) has φ = 96:2:2 and *ρ* ~3.3 g cm^-3^, whereas the Gr-coated one (**b**) has φ = 99.5:0:0.5 and *ρ* ~3.9 g cm^-3^. The Gr-coated electrode with φ = 96:2:2 and *ρ* ~3.3 g cm^-3^ is also compared (**c**).


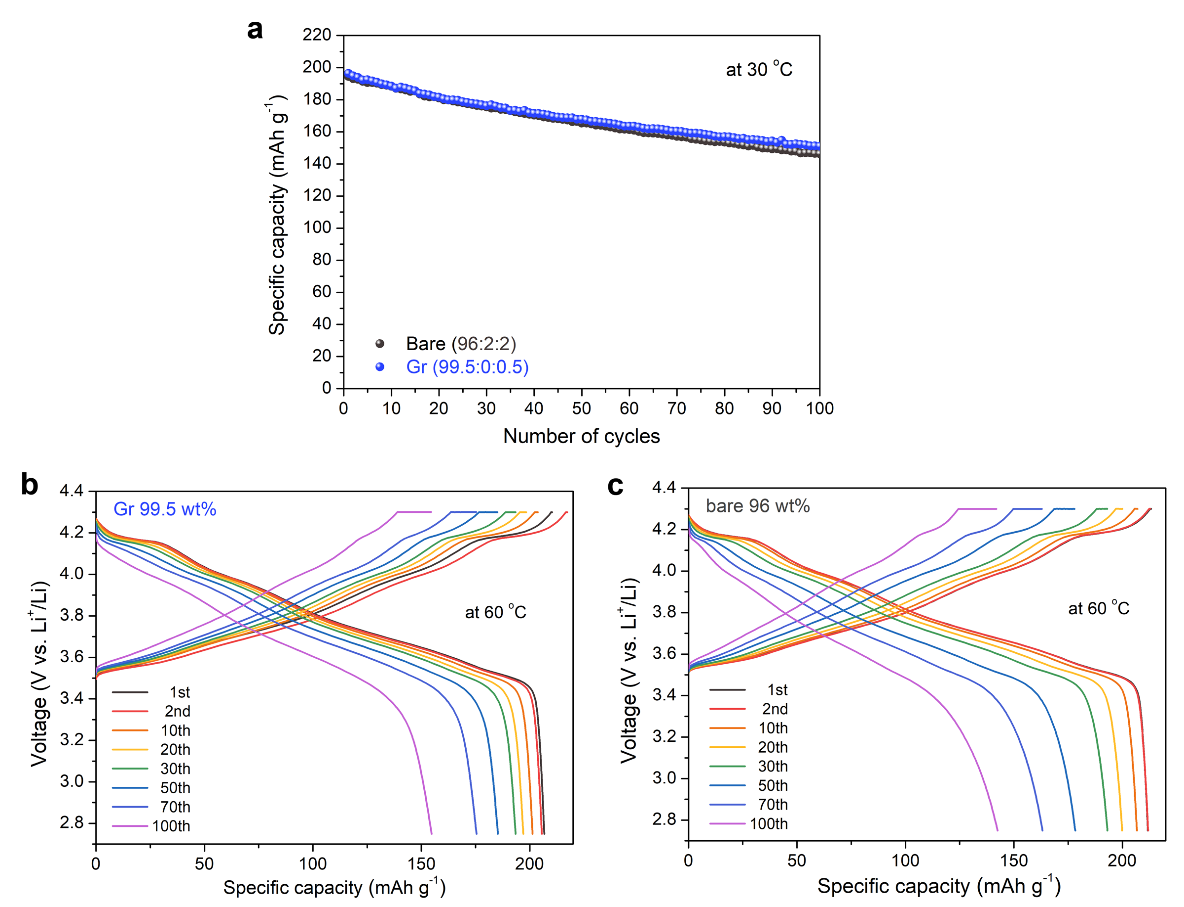


**Supplementary Fig. 20| Cycle performance of bare NCA (φ = 96:2:2/*ρ* ~3.3 g cm^-3^) and Gr-coated NCA (φ = 99.5:0:0.5/*ρ* ~3.9 g cm^-3^) electrodes**. **a**, Discharge capacity retention at 30 °C. **b**, Voltage profiles at 60 °C (**b**,**c**). The cycling tests at 30 °C and 60 °C were conducted in the voltage range of 2.75-4.3 V vs. Li^+^/Li at 0.5C.





**Supplementary Fig. 21|** Nyquist plots of the bare and Gr-coated NCA electrodes before (top) and after 100 cycles (bottom) at 60 °C.


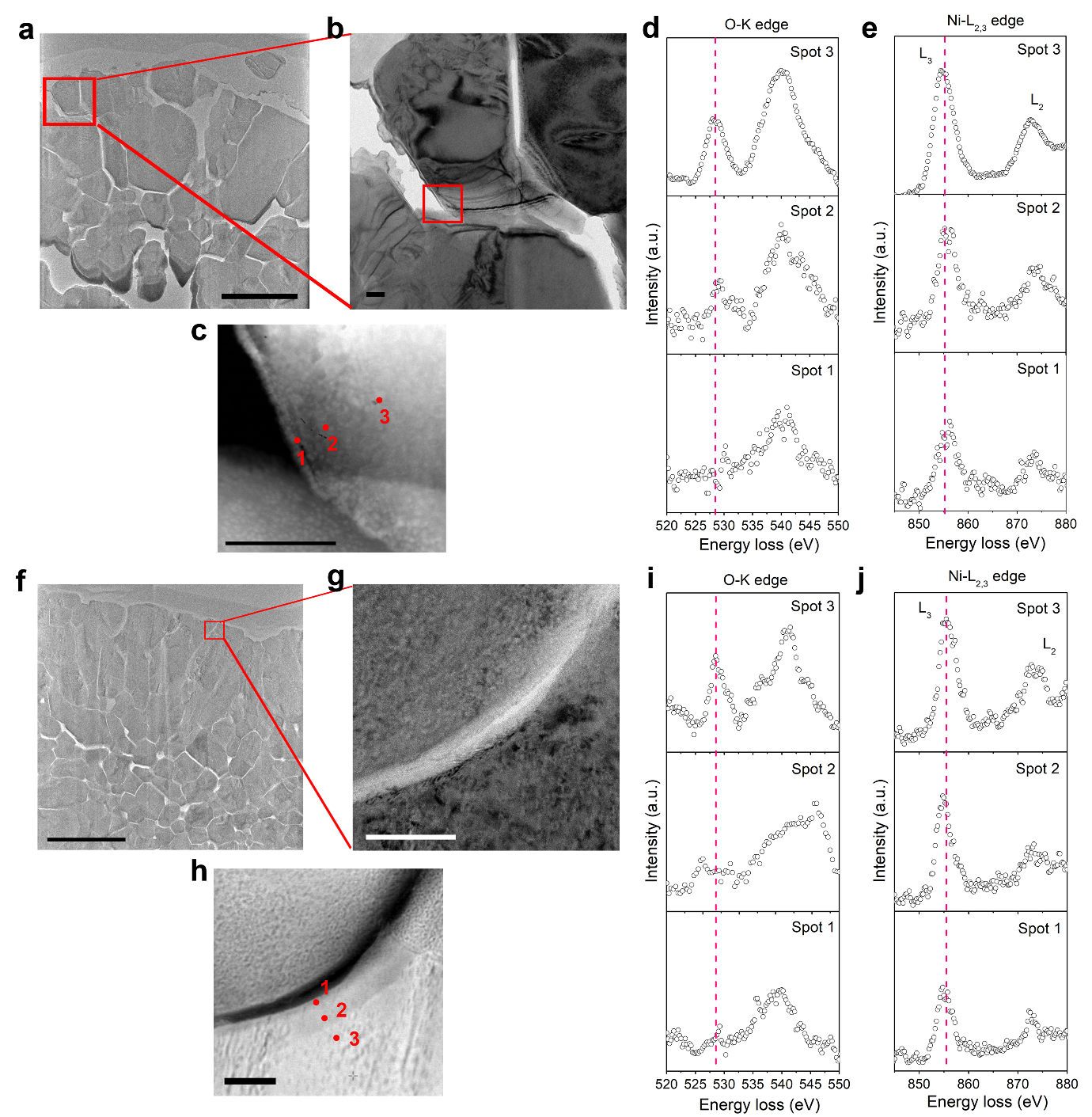


**Supplementary Fig. 22|** TEM images (**a**-**c**, **f**-**h**) and EELS profiles of O-K (**d**,**i**) and Ni-L_2,3_ (**e**,**j**) edges for the bare NCA (**a**-**e**) and Gr-coated NCA (**f**-**j**) electrodes after 100 cycles at 60 °C. The samples were prepared using a FIB of the corresponding particles. The scale bars in **a**,**f** and **b**,**c**,**g**,**h** indicate 2 μm and 100 nm, respectively. The EELS profiles of spots 1-3 were acquired from marks 1-3 in **c** and **h**. Both the samples have a NiO (Fm3m)-like surface structure (spot 1-2), while showing the LiNiO_2_ with the R3m structure in the inner region (spot 3). It should be noted that the Gr-coated particle (**f**) appears to be relatively intact, as compared to the bare one showing local microcracks (**a**).

**
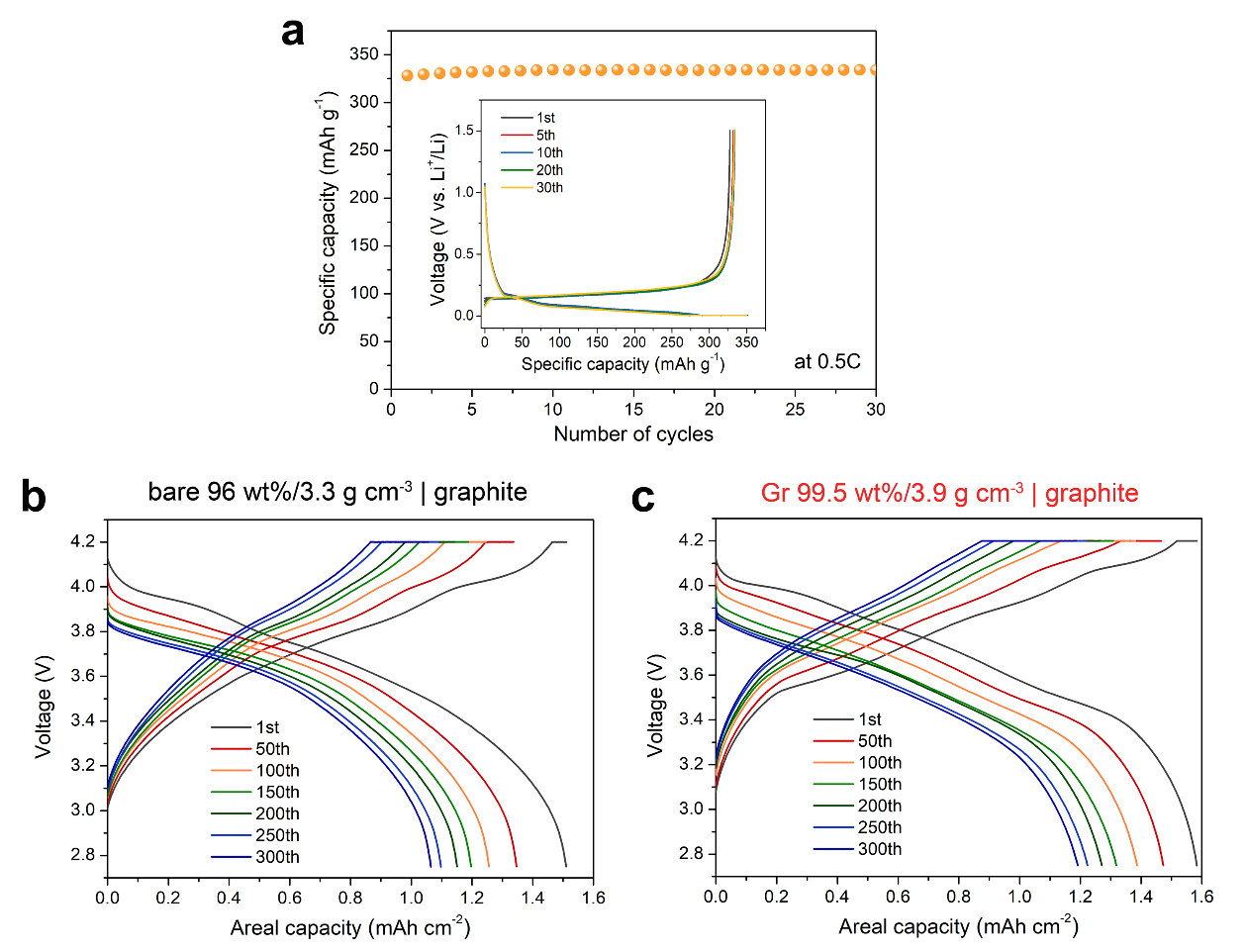
**

**Supplementary Fig. 23| Cycle performance of the anode half-cell (Li|graphite) (a) and full-cell (graphite|NCA) (b,c)**. The graphite anode half-cell was cycled at 30 °C in the voltage range of 0.005-1.5 V vs. Li^+^/Li at a current rate of 0.5C. The full-cells were cycled at 30 °C in the voltage range of 2.75-4.2 V at 0.5C.


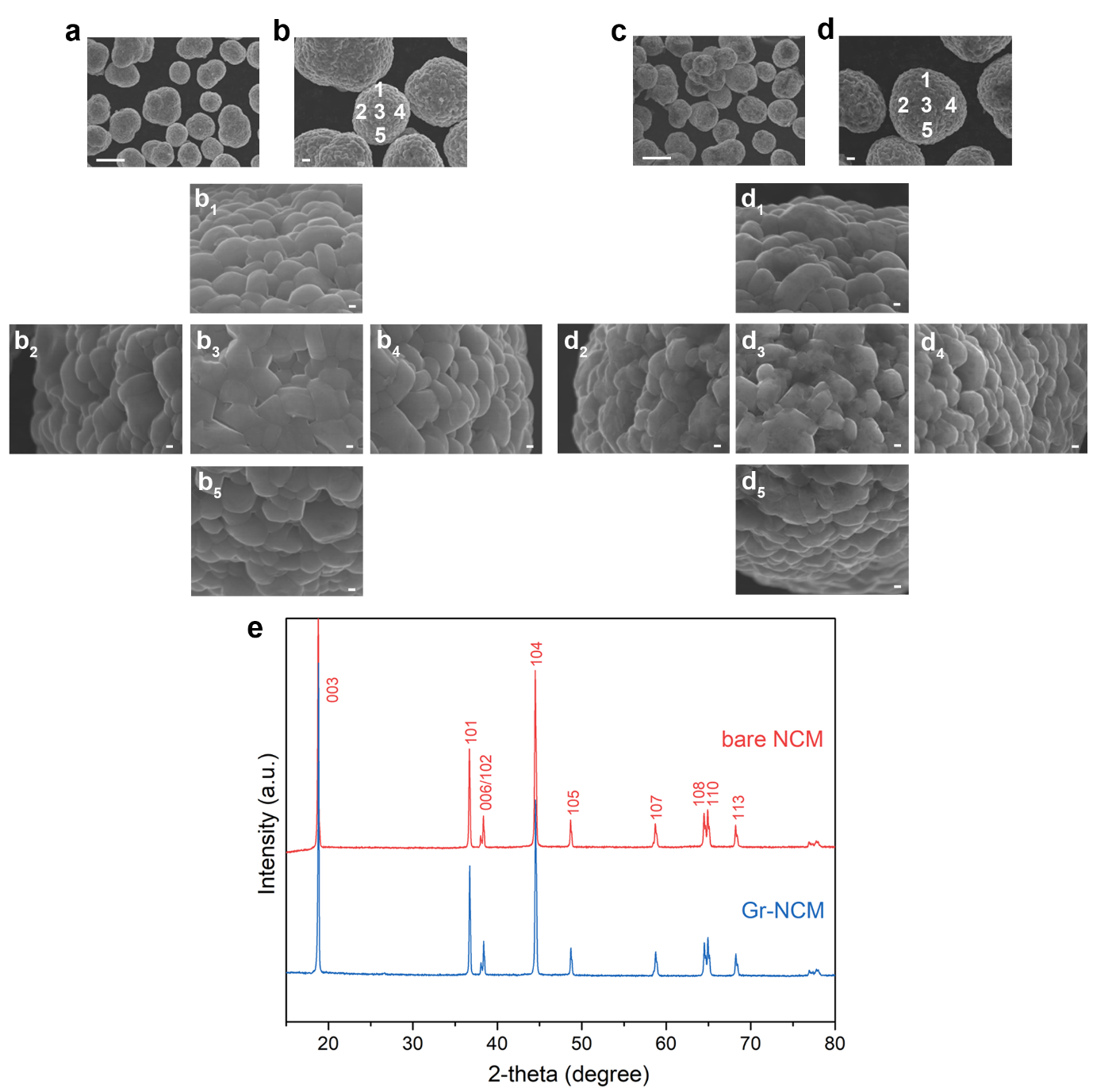


**Supplementary Fig. 24|** SEM images (**a**-**d**) and XRD patterns (**e**) of bare (**a**,**b**) and Gr-coated (thrice) (**c**,**d**) NCM811 particles. The scale bars in **a**,**c** and **b**,**d** indicate 10 μm and 1 μm, respectively, and those in **b_1_**-**b_5_** and **d_1_**-**d_5_** indicate 100 nm.


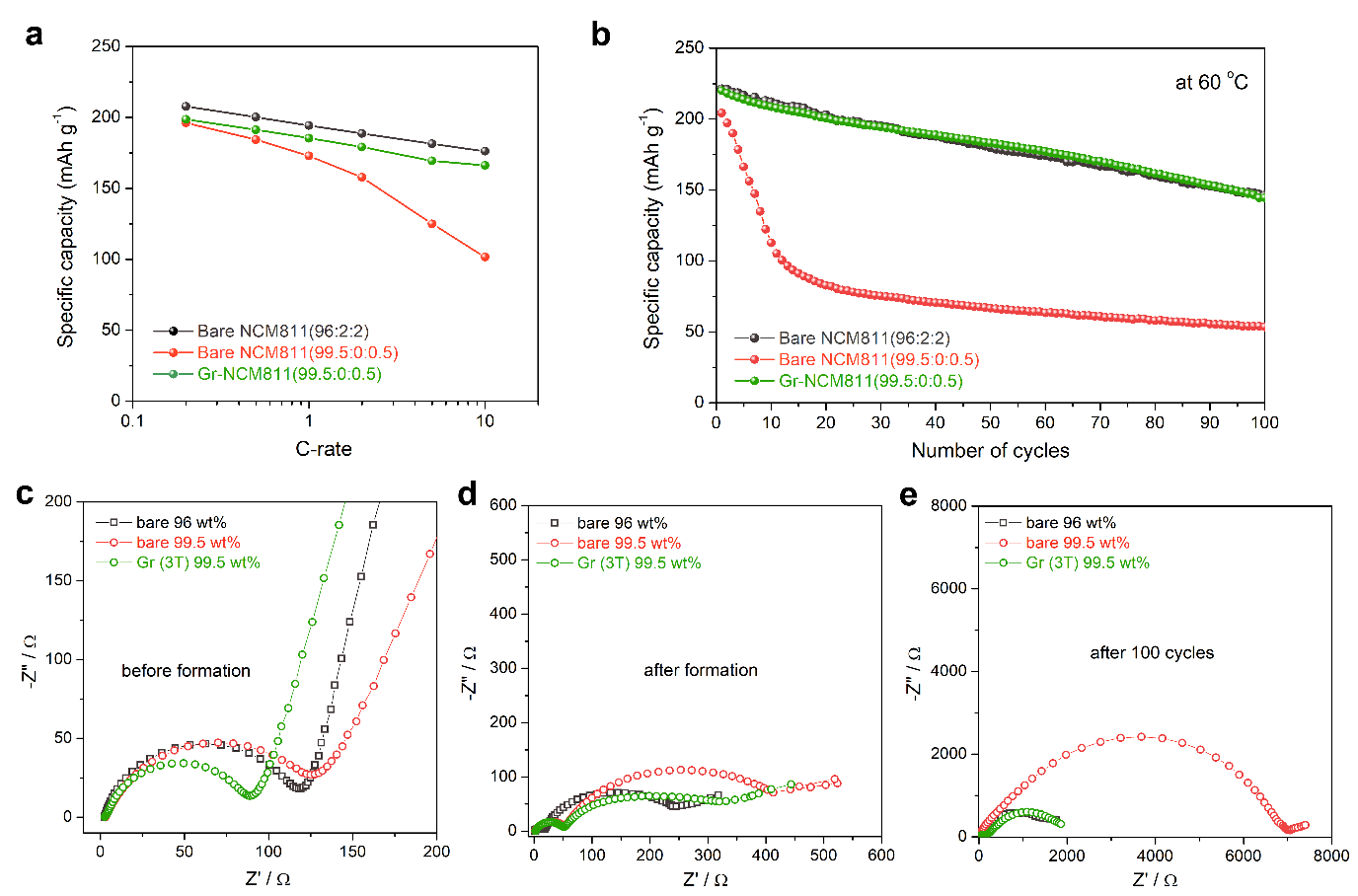


**Supplementary Fig. 25| Electrochemical properties of bare (φ = 96:2:2/*ρ* ~3.3 g cm^-3^) and Gr-coated (φ = 99.5:0:0.5/*ρ* ~3.9 g cm^-3^) NCM811 cathodes.** **a**, Rate capability at 0.2-10C. **b**, discharge capacity retention, measured at 60 °C in the voltage range of 2.75-4.3 V vs. Li^+^/Li at 0.5C. **c**-**e**, Nyquist plots measured before formation, after formation, and after 100 cycles. For comparison, the bare electrode with electrode parameters of φ = 99.5:0:0.5 and *ρ* ~3.9 g cm^-3^ was evaluated.

**
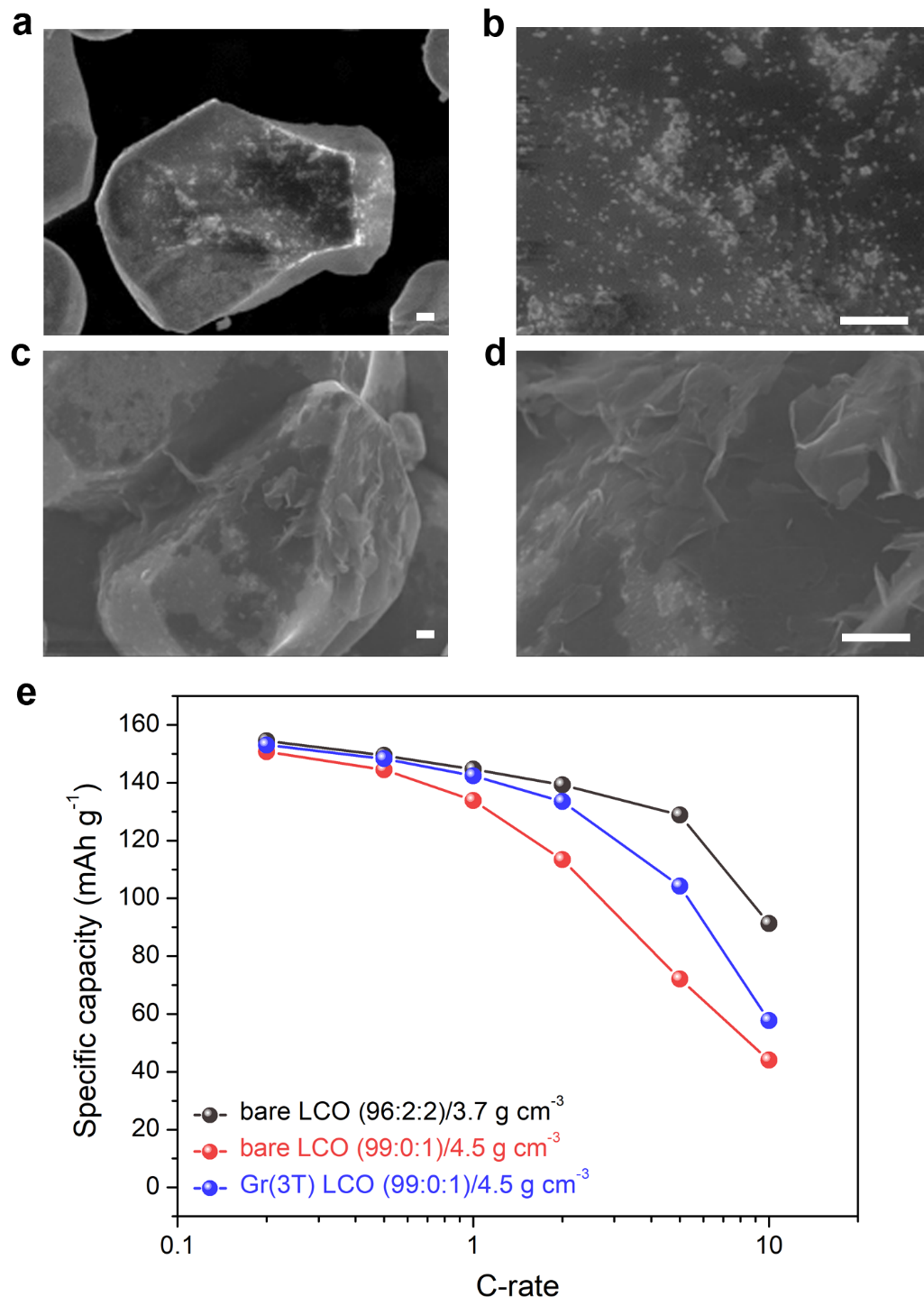
**

**Supplementary Fig. 26| Surface morphology and electrochemical properties of bare and Gr-coated LiCoO_2_ (LCO) cathodes**. **a**-**d**, SEM images of the bare (**a**,**b**) and Gr-coated (thrice) (**c**,**d**) LCO particles. **e**, Rate performance of the bare and Gr-coated LCO electrodes, measured in the voltage range of 2.75-4.3 V vs. Li^+^/Li at 0.2-10C. The electrode composition and density are shown. The Gr coating coverage on LCO is found to be relatively poor, as compared to Ni-rich oxides, which results in inferior rate capability at high C-rates even for a high electrode density of 4.5 g cm^-3^. The scale bars in a-d indicate 1 μm.


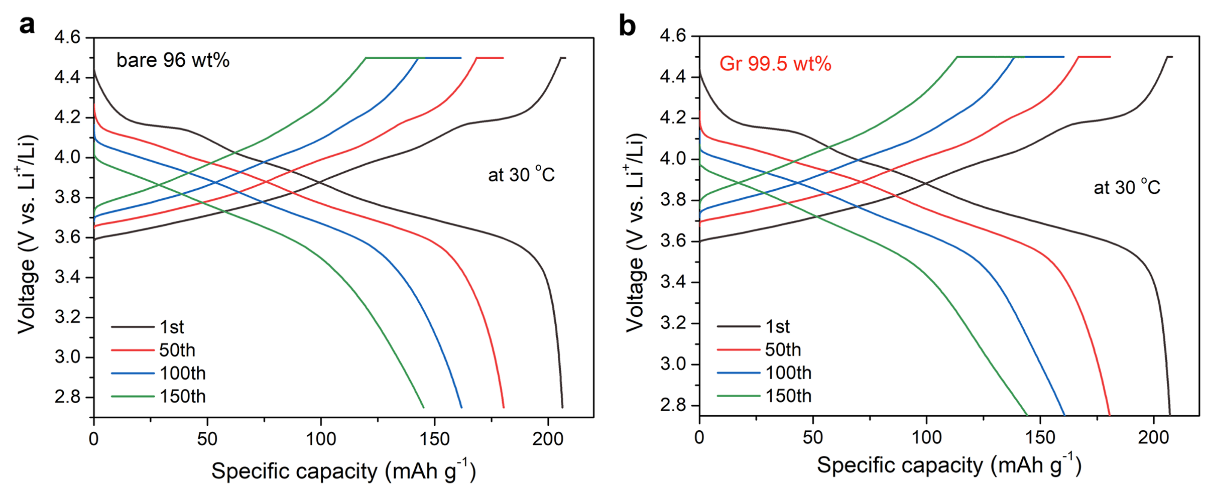


**Supplementary Fig. 27|** Voltage profiles of bare (**a**) and Gr-coated (**b**) NCA cathodes, measured at 30 °C in the voltage range of 2.75-4.5 V vs. Li^+^/Li at 0.5C.


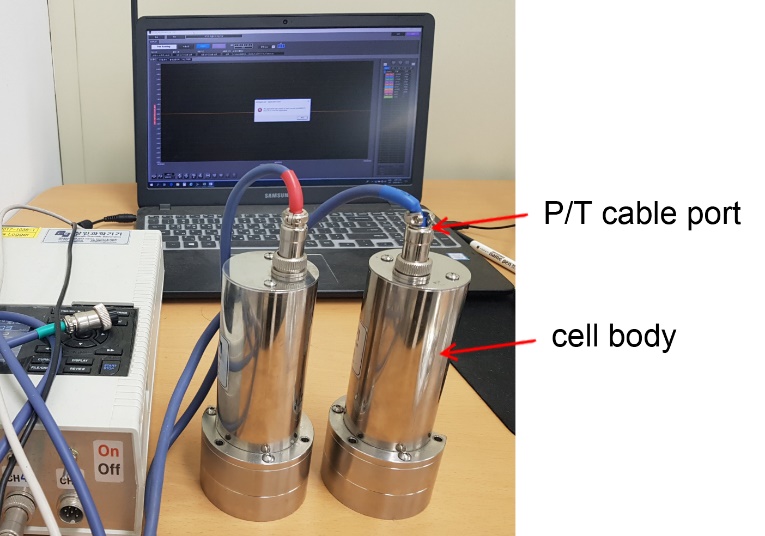


**Supplementary Fig. 28|** Digital picture of a home-made gas-cell system.


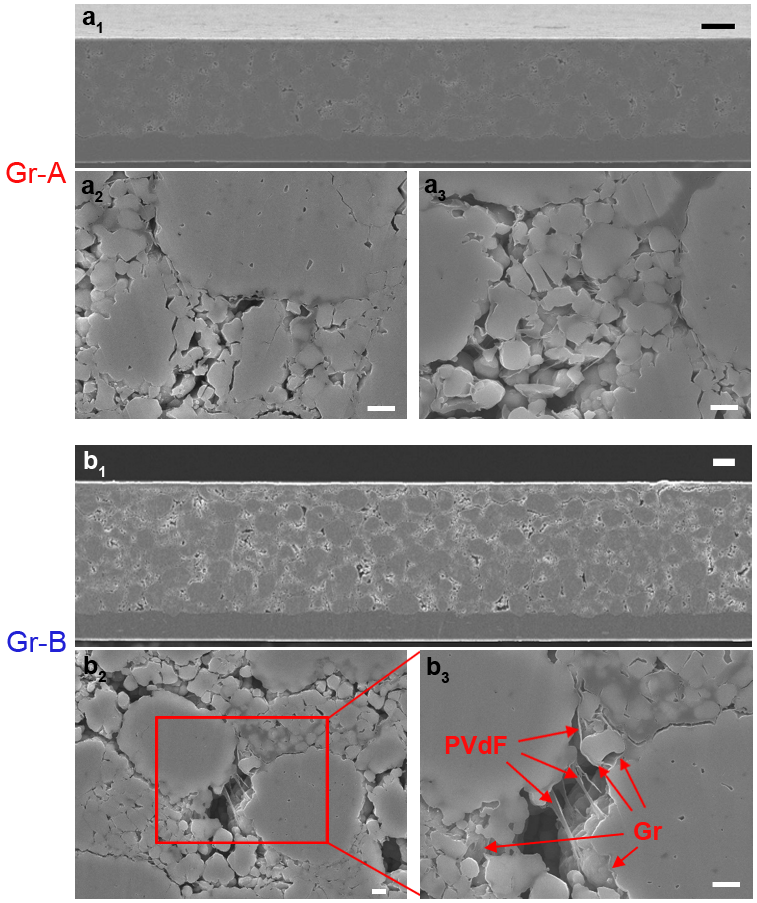


(continued)


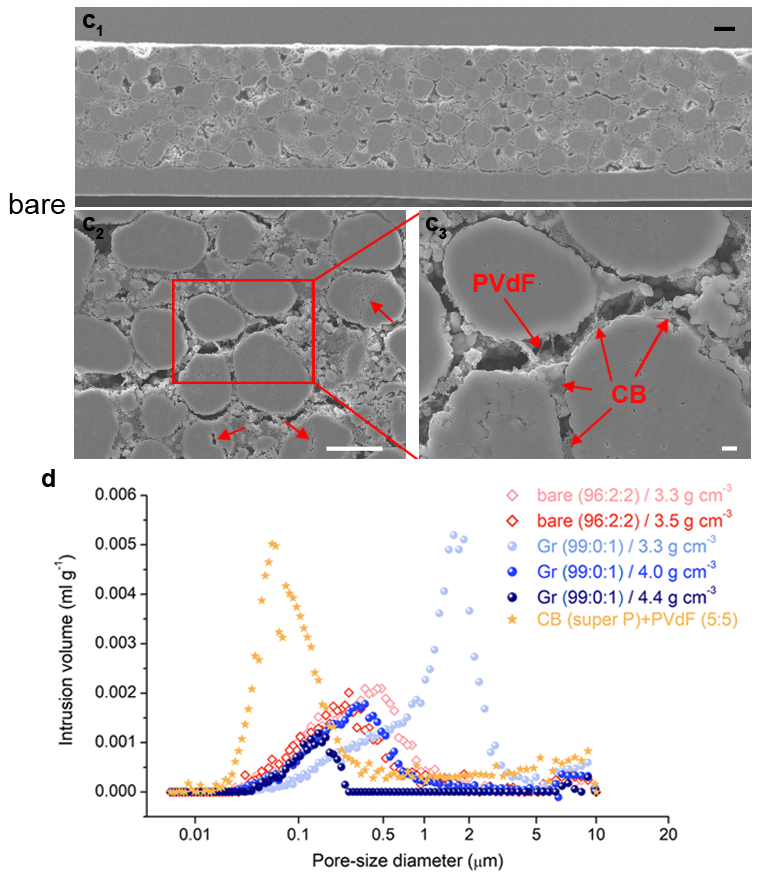


**Supplementary Fig. 29|** Cross-sectional SEM images (**a**-**c**) and pore-size distributions (**d**) of bare and Gr-coated NCA cathodes with different electrode parameters. The pore-size distributions are well-matched with the electrode design parameters. The scale bars in **a_1_**, **b_1_**, **c_1_** and **c_2_** indicate 10 μm, and those in **a_2_**, **a_3_**, **b_2_**, **b_3_**, and **c_3_** are 1 μm. The inactive components, such as PVdF, CB, and Gr nanosheets are observed between NCA particles. For the bare electrode, intra-particle cracking is notable as highlighted by red arrows (**c_2_**).


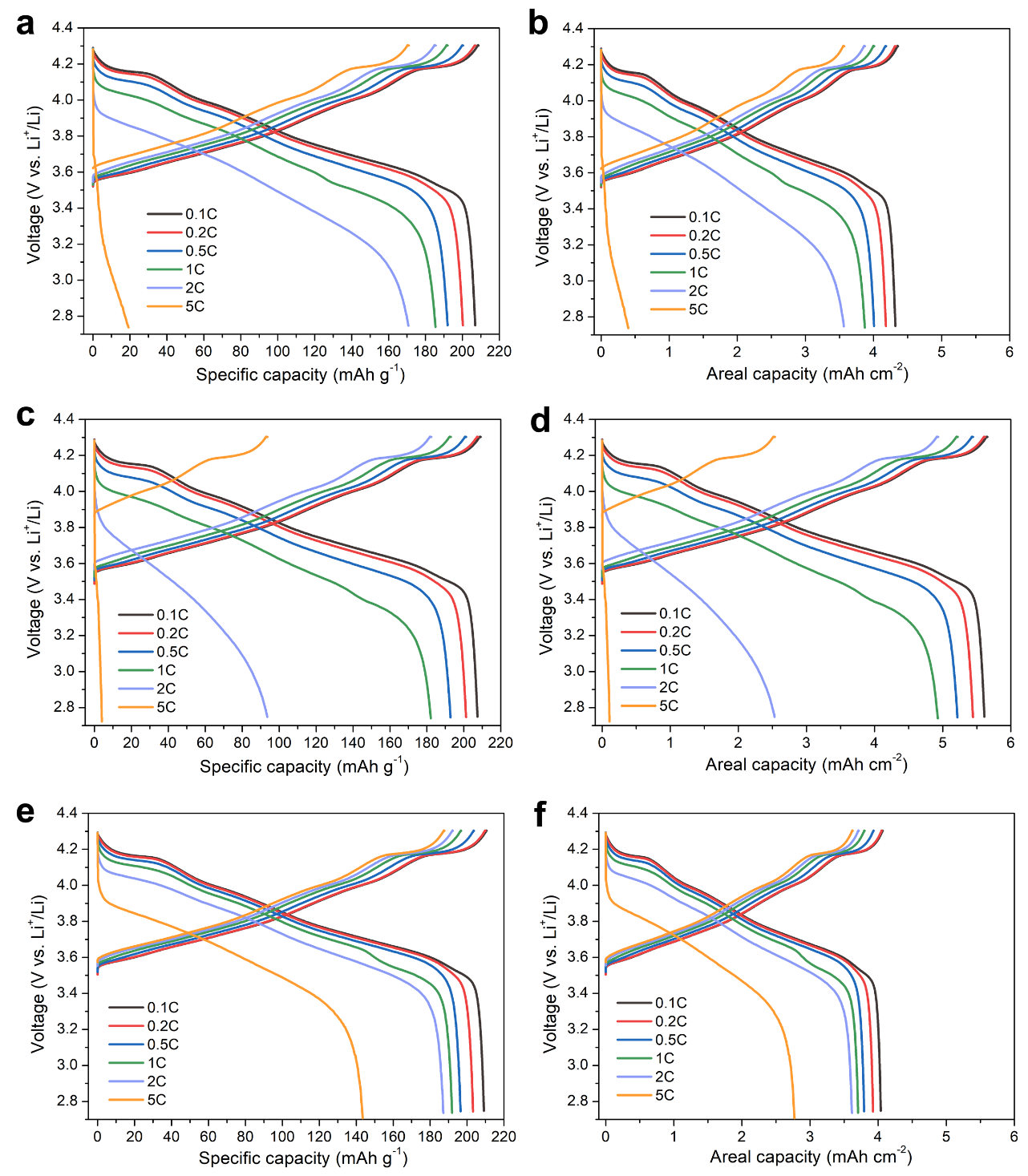


**Supplementary Fig. 30 | Voltage profiles of the bare and Gr-coated electrodes with different electrode parameters, showing the specific capacities and areal capacities measured at different discharge current rates (0.1-5C)**. The Gr-coated electrodes commonly have φ=99:0:1 and *ρ*~4.3 g cm^-3^; however, the Gr-A (**a**,**b**) and Gr-B (**c**,**d**) electrodes have ~20 mg cm^-2^ and ~27 mg cm^-2^ in m_areal_, respectively; on the other hand, the bare electrode (**e**,**f**) has m_areal_~ 20 mg cm^-2^, φ=96:2:2, and *ρ*~3.3 g cm^-3^. All the electrodes were charged at a current rate of 0.1C with a CC-CV mode using 0.05C cut-off.


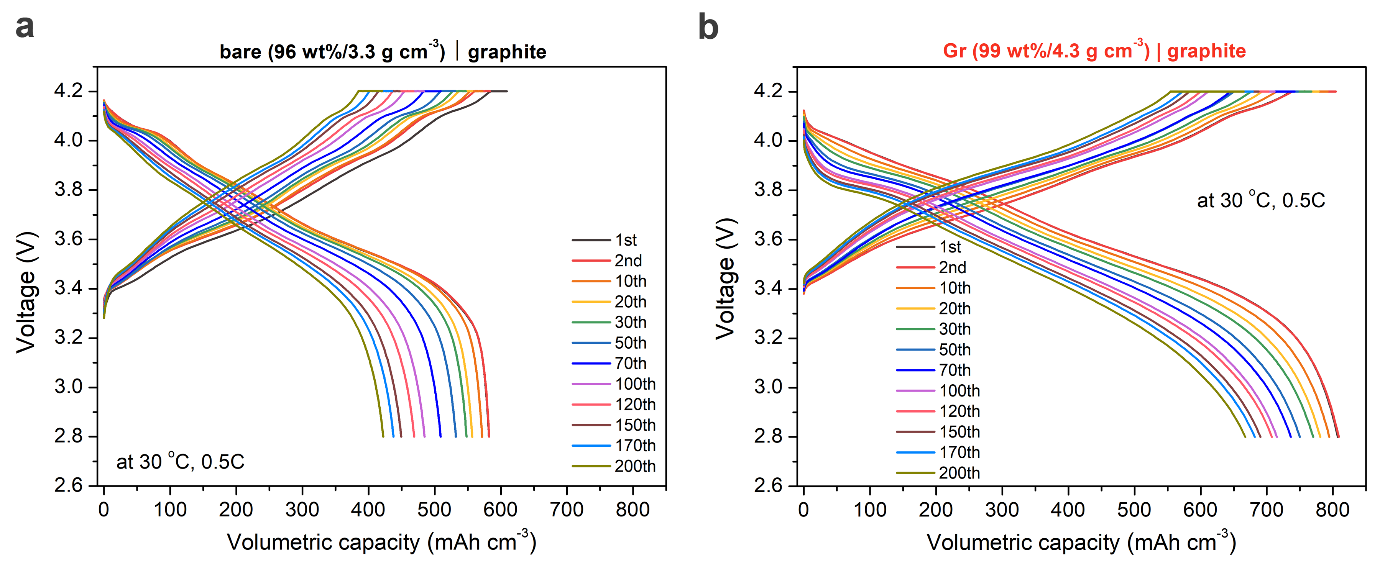


**Supplementary Fig. 31| Cycle performance of the full-cells (graphite|NCA) using the bare cathode (a) and Gr-coated NCA cathodes (b)**. The m_areal_ of the NCA cathodes was ~17 mg cm^-2^. The full-cells were cycled at 30 °C in the voltage range of 2.75-4.2 V at 0.5C.

**Supplementary Table S1**. Literature comparison of the electrode parameters and measured capacities of Gr-coated cathodes for LIBs.

| **Active**  **materials** | **Gr type**  **(fabrication method)** | **Coating**  **method** | **Electrode composition** | **Mass loading**  **[mg cm^-2^]** | **Electrode density**  **[g cm^-3^]** | **Specific capacity**  **[mAh g^-1^]** | **Q_areal_**  **(or Q_vol_)** | **Ref.** |
| --- | --- | --- | --- | --- | --- | --- | --- | --- |
| LiNi_0.8_Co_0.15_Al_0.05_O_2_ | Electrochem.  exfoliated Gr | Immersion  (amphiphilic surfactants) | AM(Gr):CB:PVDF  =98.5(0.5):0:1 | ~27 | ~4.3 | 201@0.2C | Q_areal_~5.4 mAh cm^-2^  ;Q_vol_~860 mAh cm^-3^  @0.2C | **Our work** |
| LiNi_0.8_Co_0.15_Al_0.05_O_2_ | Solution-exfoliated Gr | Pickering emulsion (acetonitrile/hexane) | AM(Gr):CB:PVDF  =98.7(0.5):0:0.8 | ~11 | ~3.6 | ~188@0.1C | Q_areal_~2.07 mAh cm^-2^  ;Q_vol_~675 mAh cm^-3^  @0.1C | 7 |
| LiNi_0.8_Co_0.15_Al_0.05_O_2_ | Gr nanodot  (electrochem.) | Immersion  (ethanol) | AM(Gr):CB:PVDF  =79.6(0.4):10:10 | 1.4 | N/A | 195@0.1C | Q_areal_~0.27 mAh cm^-2^ @0.1C | 8 |
| LiNi_0.6_Co_0.1_Mn_0.3_O_2_ | Gr balls (CVD) | Nobilta milling | AM(Gr):CB:PVDF  =97.0(1.0):0.5:1.5 | ~25 | ~4.1 | ~196@0.1C | Q_areal_~4.75 mAh cm^-2^  ;Q_vol_~760 mAh cm^-3^  @0.1C | 9 |
| LiNi_0.6_Co_0.2_Mn_0.2_O_2_ | rGO  (Hummer’s) | Immersion (APTES/toluene) | AM(Gr):CB:PVDF  =95.5(0.5):2:2 | N/A | N/A | ~199@0.1C | N/A | 10 |
| LiCo_1/3_Ni_1/3_Mn_1/3_O_2_ | rGO  (Hummer’s) | Spray drying  (DI water) | AM(Gr):CB:PVDF  =72.8(7.2):10:10 | 3.4-3.6 | N/A | 224@0.2C | Q_areal_~0.76 mAh cm^-2^  @0.2C | 11 |
| Li(Li_0.1_Al_0.1_Mn_1.8_)O_4_ | Gr (ball mill of graphite) | High-energy  ball-mill | AM:graphite:CB:PVDF  =80:7:7:6 | N/A | N/A | ~100@1C | N/A | 12 |
| LiNi_0.5_Mn_1.5_O_4_ | rGO  (Hummer’s) | Immersion  (ethanol) | AM(Gr):CB:PVDF  =76.2(3.8):10:10 | 2-3 | N/A | ~113@0.2C | Q_areal_~0.23 mAh cm^-2^ @0.2C | 13 |
| LiFePO_4_ | GO  (Hummer’s) | Spray drying  (DI water) | AM(Gr):CB:PVDF  =72.8(7.2):15:5 | 3-4 | N/A | 148@0.1C | Q_areal_~0.44 mAh cm^-2^ @0.1C | 14 |
| LiFePO_4_ | rGO  (Hummer’s) | Immersion  (CTAB/DI water) | AM(Gr):CB:PTFE  =72.8(7.2):10:10 | N/A | N/A | ~150@0.1C | N/A | 15 |

**Supplementary References**

1. Parvez, K. et al. Exfoliation of graphite into graphene in aqueous solutions of inorganic salts. *J. Am. Chem. Soc.* **136**, 6083−6091 (2014).
2. Tian, R. et al. Quantifying the effect of electronic conductivity on the rate performance of nanocomposite battery electrodes. *ACS Appl. Energy Mater.* **3**, 2966–2974 (2020).
3. McLachlan, D. S., Blaszkiewicz, M. &Newnham, R. E. Electrical Resistivity of Composites. *J. Am. Ceram. Soc.* **73**, 2187-2203 (1990).
4. Cernescu, A. et al. Label-Free Infrared Spectroscopy and Imaging of Single Phospholipid Bilayers with Nanoscale Resolution Phospholipid Bilayers with Nanoscale. *Anal. Chem.* **90**, 10179-10186 (2018).
5. Kiwi, J., & Nadtochenko, V. Evidence for the Mechanism of Photocatalytic Degradation of the Bacterial Wall Membrane at the TiO_2_ Interface by ATR-FTIR and Laser Kinetic Spectroscopy. *Langmuir* **21**, 4631-4641 (2005).
6. Krishna, R. et al. Facile synthesis of hydrogenated reduced graphene oxide via hydrogen spillover mechanism. *J. Mater. Chem.* **22**, 10457 (2012).
7. Park, K.Y. et al. Concurrently Approaching Volumetric and Specific Capacity Limits of Lithium Battery Cathodes via Conformal Pickering Emulsion Graphene Coatings. *Adv Energy Mater*. **10**, 2001216 (2020).
8. He, X. et al. Improved Electrochemical Performance of LiNi_0.8_Co_0.15_Al_0.05_O_2_ Cathode Material by Coating of Graphene Nanodots. *J. Electrochem. Society* **166**, A1038-A1044 (2019).
9. Son, I.H. et al. Graphene balls for lithium rechargeable batteries with fast charging and high volumetric energy densities. *Nat Commun* **8**, 1561 (2017).
10. Shim, J.-H., Kim, Y.-M., Park, M., Kim, J. & Lee, S. Reduced Graphene Oxide-Wrapped Nickel-Rich Cathode Materials for Lithium Ion Batteries. *ACS Appl. Mater. Interfaces* **9**, 18720-18729 (2017).
11. He, J.-r. et al. Synthesis and electrochemical properties of graphene-modified LiCo_1/3_Ni_1/3_Mn_1/3_O_2_'cathodes for lithium ion batteries. *RSC Adv.* **4**, 2568-2572 (2014).
12. Noh, H.K., Park, H.-S., Jeong, H.Y., Lee, S.U. & Song, H.-K. Doubling the Capacity of Lithium Manganese Oxide Spinel by a Flexible Skinny Graphitic Layer. *Angew. Chem Int Ed.* **53**, 5059-5063 (2014).
13. Fang, X., Ge, M., Rong, J. & Zhou, C. Graphene-oxide-coated LiNi_0.5_Mn_1.5_O_4_ as high voltage cathode for lithium ion batteries with high energy density and long cycle life. *J. Mater. Chem A* **1**, 4083 (2013).
14. Zhou, X., Wang, F., Zhu, Y. & Liu, Z. Graphene modified LiFePO4 cathode materials for high power lithium ion batteries. *J. Mater. Chem* **21**, 3353-3358 (2011).
15. Wei, W. et al. The effect of graphene wrapping on the performance of LiFePO_4_ for a lithium ion battery. *Carbon* **57**, 530-533 (2013).
